# A Structural Systems Biology Approach for Quantifying the Systemic Consequences of Missense Mutations in Proteins

**DOI:** 10.1371/journal.pcbi.1002738

**Published:** 2012-10-18

**Authors:** Tammy M. K. Cheng, Lucas Goehring, Linda Jeffery, Yu-En Lu, Jacqueline Hayles, Béla Novák, Paul A. Bates

**Affiliations:** 1 Biomolecular Modelling Laboratory, Cancer Research UK London Research Institute, London, United Kingdom; 2 Max Planck Institute for Dynamics and Self-Organization, Göttingen, Germany; 3 Cell Cycle Laboratory, Cancer Research UK London Research Institute, London, United Kingdom; 4 Computer Laboratory, University of Cambridge, Cambridge, United Kingdom; 5 Department of Biochemistry, University of Oxford, Oxford, United Kingdom; National Institutes of Health, United States of America

## Abstract

Gauging the systemic effects of non-synonymous single nucleotide polymorphisms (nsSNPs) is an important topic in the pursuit of personalized medicine. However, it is a non-trivial task to understand how a change at the protein structure level eventually affects a cell's behavior. This is because complex information at both the protein and pathway level has to be integrated. Given that the idea of integrating both protein and pathway dynamics to estimate the systemic impact of missense mutations in proteins remains predominantly unexplored, we investigate the practicality of such an approach by formulating mathematical models and comparing them with experimental data to study missense mutations. We present two case studies: (1) interpreting systemic perturbation for mutations within the cell cycle control mechanisms (G2 to mitosis transition) for yeast; (2) phenotypic classification of neuron-related human diseases associated with mutations within the mitogen-activated protein kinase (MAPK) pathway. We show that the application of simplified mathematical models is feasible for understanding the effects of small sequence changes on cellular behavior. Furthermore, we show that the systemic impact of missense mutations can be effectively quantified as a combination of protein stability change and pathway perturbation.

## Author Summary

Small changes in protein sequences, such as missense mutations resulting from genetic variations in the genome, can have a large impact on cellular behavior. Consequently, numerous studies have been carried out to evaluate the disease susceptibility of missense mutations by directly analyzing their structural or functional impact on proteins. Such an approach has been shown to be useful for inferring the likelihood of a mutation to be disease-associated. However, there are still many unexplored avenues for improving disease-association studies, due to the fact that the dynamics of biological pathways are rarely considered. We therefore explore the practicality of a structural systems biology approach, combining pathway dynamics with protein structural information, for projecting the physiological outcomes of missense mutations. We show that stability changes of proteins due to missense mutations and the sensitivity of a protein in terms of regulating pathway dynamics are useful measures for this purpose. Furthermore, we demonstrate that complicated mathematical models are not a prerequisite for mapping protein stabilities to network perturbation. Thus it may be more feasible to study the systemic impact of missense mutations associated with complex pathways.

## Introduction

How one links genetic information to physiological outcomes is an important issue in the current ‘post-GWAS’ (genome-wide association studies) era [1]. One specific topic regarding this problem is the functional annotation of non-synonymous single nucleotide polymorphisms (nsSNPs) that cause amino acid changes in proteins. However, the difficulty of annotating nsSNPs has slowed down the pace of investigating their molecular consequences. Therefore, as the speed of identifying new SNPs is high, there is now a distinct sense of urgency to resolve this problem – an immediate focus is the 1000 Genomes Project (http://www.1000genomes.org/) that has identified approximately 100,000 nsSNPs in need of further analyses. Indeed, the urgent requirement for SNP annotation has also motivated the CAGI experiment (Critical Assessment of Genome Interpretation; http://genomeinterpretation.org/) that encourages community-wide efforts in predicting the phenotypic impacts of genome variation.

Interpreting the physiological effect on cells due to missense mutations in proteins is not a simple task. This is partly achievable through analyzing the increasing number of protein structures deposited in the Protein Data Bank (http://www.rcsb.org/) and through functional annotation of proteins [2]. Investigating protein structures allows for a qualitative view of pathway dynamics; a more quantitative approach is to use mathematical modeling. Indeed, our understanding of cellular behavior during the last two decades has been significantly improved through the application of mathematical modeling methods such as ordinary differential equations (ODE) and rule-based simulations [3], [4], [5], [6], [7], [8]. However, the idea of integrating the dynamical aspects of proteins and their associated pathways to investigate the systemic impact of missense mutations is still in an early stage of development. In 2007 Stein et al. proposed the idea of integrating structural and pathway information for estimating key kinetic constants associated with biochemical pathways [9]. More recently, Kiel and Serrano [10] studied how missense mutations in the Ras-binding domain of c-Raf (RafRBD) affect the expression of the downstream protein Erk by investigating the structure of RafRBD and constructing an ODE model describing Erk signaling pathway [10].

The work of Kiel and Serrano suggests that integrating protein structural analysis with pathway modeling can be a useful method to facilitate the physiological annotation of missense mutations in proteins. However, the effectiveness of this approach at quantifying missense mutations located in different proteins remains unexplored. Also unexplored is the utility of this approach with simpler mathematical models, considering only the dynamics of key proteins while the remaining proteins in the pathway are omitted – this is potentially a more practical approach for achieving an improved inference of the parameter space, thereby increasing the reliability of the analysis (current ODE models describing biological pathways often contain tens or hundreds of parameters that can neither be easily measured nor calibrated experimentally). Furthermore, extensive investigation is required to determine how the approach performs when annotating missense mutations whose physiological outcomes can be clinically defined and examined.

These issues are discussed in this work by gauging the systemic impacts of missense mutations through integrating protein and pathway behavior via reduced ODE models. Here we present and discuss the measurement of a ‘systemic impact factor’ (SIF), defined as a function of free energy change (ΔΔG) and systemic control (C^S^_pi_, see Methods section ‘Control coefficient’), as a practical approach for evaluating the relative effects of missense mutations in a specific system. For mutations appearing in proteins whose complexed and uncomplexed states are both considered in the model, we calculate their maximum SIFs by taking the maximum ΔΔG between the two states. This is because the average score of the two protein states does not necessarily have a clear biophysical meaning in terms of describing the overall stability change of a mutation. Although summing the ΔΔGs calculated in the two protein states may have biophysical meaning, complications will be incurred when comparing the SIFs to other proteins that only have one conformational state analyzed in the model (either complexed or uncomplexed). Therefore, by using the maximum ΔΔGs we do not compromize the biophysical meaning of SIF and at the same time make the SIF scores more comparable across different proteins that may or may not have two states.

The benchmark includes two biological systems: (1) the fission yeast G2 to Mitosis (G2-M) transition and (2) the human MAPK signaling pathway. The first system is a well-defined system for studying the genotype-phenotype relationship as the systemic perturbation of missense mutations can be directly benchmarked to the length change of yeast cells. We use the temperature-sensitive yeast strains as experimental models, each of them containing a single missense mutation in protein Cdk1 or Cyclin B (CycB), and we measure their cell lengths at septation (septation is immediately followed by mitosis). Finally, the practicality of the SIF score in quantifying the systemic effect of missense mutations is evaluated by the correlation between the calculated SIF scores and *in vivo* cell lengths. The second benchmark system represents a more complex example in which the target mutations are spread within four different proteins (H-Ras, Raf-1, Braf and Me) and lead to clinical symptoms (in this case the neuro-cardio-facial-cutaneous syndrome) that have different prognoses and risk of complication. To determine whether or not a simple ODE model can be used to infer the systemic perturbation of missense mutations, we construct a reduced ODE model that includes only 12 parameters for the calculation of SIF values. We then place the mutations into subgroups according to their predicted SIF scores, and record whether our classification reveals the underlying difference between disease mechanisms.

## Results

### *In silico* model of the G2-M transition in yeast

**Figure 1:**
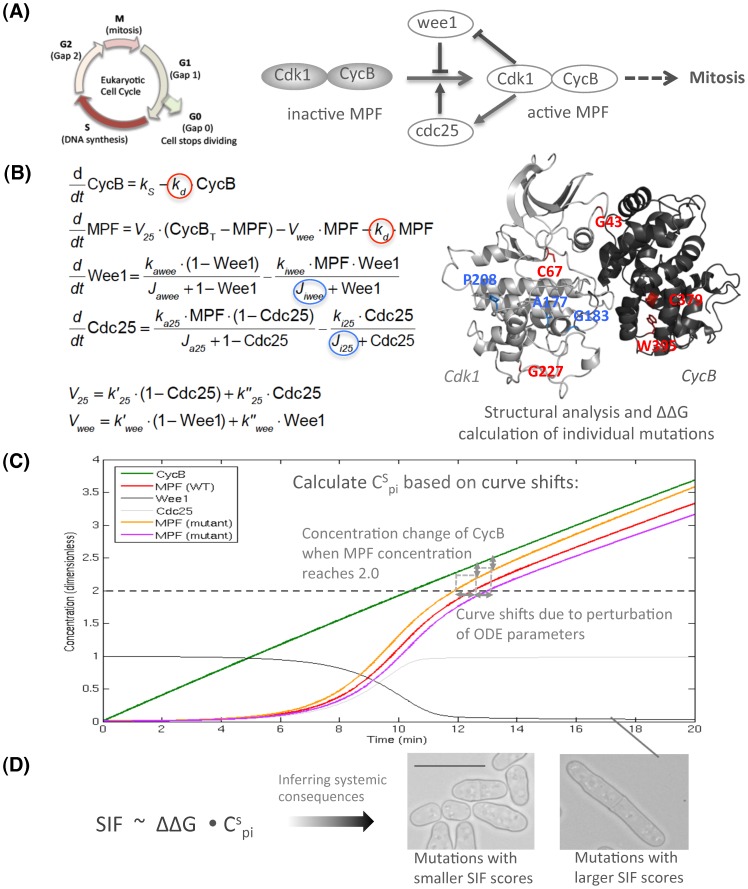
The procedure of calculating SIF scores. (A) Identifying the target system for study. In this case we show the scheme of the G2-M model that regulates the G2 to mitosis transition in the cell cycle. (B) Mapping mutations onto their 3D structures (Cdk1 and CycB in this example) and associating them with the ODE parameters. Mutations located at or close to the active site (colored in blue) are considered to perturb the ODE rate constants that describe interactions between MPF and their regulating kinases wee1 and cdc25 (shown with blue circles). Mutations that are not in the functional sites (colored in red) are considered to perturb the ODE rate constants describing the rate of protein degradation (shown with red circles). Also, for each mutation we evaluate its ΔΔG that is considered as the perturbation of ODE parameters. (C) Calculating the C^S^_pi_ that reflects the sensitivity of perturbing ODE parameters in terms of regulating the downstream reporter protein (MPF in the G2-M model). Here we show the perturbation on the degradation rate of MPF as an example: The green arrows mark the effect of perturbation on CycB concentration when cells enter mitosis, which is a result of MPF curve shifts (the red line represents wild type whereas orange and purple lines are mutant types). (D) Inferring the systemic consequences of mutations based on ΔΔG and C^S^_pi_. Mutations that have smaller or larger SIF scores are likely to have smaller or larger sizes at septation, respectively. The scale bars shown in the microscopic photos represent the average length of wild-type yeasts.

The G2-M transition controls when a cell enters mitosis and determines the size of a cell at the point of division into two daughter cells. In fission yeast, *Schizosaccharomyces pombe*, this involves Cdk1, CycB, Wee1 and Cdc25. In the G2 phase, Cdk1 and CycB form a complex known as the mitosis promoting factor (MPF), which brings about the G2-M transition [11]. The activity of MPF is regulated by the protein kinase Wee1 [12] and the protein phosphatase Cdc25 [13], [14]: Wee1 inhibits the activity of MPF by phosphorylating Cdk1, and Cdk1 also exerts negative feedback on Wee1 by phosphorylating it. In addition, Cdc25 activates MPF by dephosphorylating Cdk1 and vice versa [15]. The Wee1-MPF-Cdc25 control system increases the ratio of active MPF over its inactive state and eventually promotes a cell into mitosis (Figure 1A).

The model we present here (Table 1) is based on the first realistic model of MPF activation published by Novak and Tyson [16]. Two basic assumptions of our model are 1) the total amount of Cdk1 (Cdk1_T_) present in the system is constant and in excess (far greater than the initial concentration of CycB) [17], and 2) all CycB forms a complex with Cdk1 immediately after it is synthesized since Cdk1 binds to CycB strongly and is in excess of CycB: that is, CycB_T_ = MPF (active form of MPF)+preMPF (inactive form of MPF).

**Table 1:** Differential equations of the G2-M model.

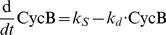
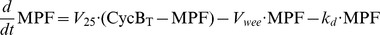
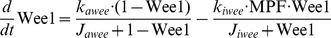
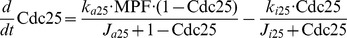
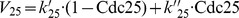
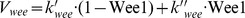
*Initial conditions (dimensionless):*
CycB = 0.01; MPF = 0.01; Wee1 = 1.0; Cdc25 = 0.01
k_S_ = 0.2; k_d_ = 0.008;
k′_25_ = 0.008; k″_25_ = 0.89; k′_wee_ = 0.03; k″_wee_ = 0.18;
k_awee_ = 0.61; k_iwee_ = 0.71; k_a25_ = 0.80; k_i25_ = 0.35
*Michaelis constants (dimensionless):*
J_awee_ = 0.90; J_iwee_ = 0.21; J_a25_ = 0.19; J_i25_ = 0.93

The parameters of the ODEs are: k_s_ is the rate of CycB synthesis and is associated with the concentration of Cdk1; k_d_ describes the degradation rate of CycB and the degradation rates of MPF. V_25_ and V_wee_ are the activation and inactivation rates of MPF, respectively. K_awee_ and K_iwee_ are the rates of Wee1 being activated by a phosphatase (which is not explicitly formulated in our model) and inactivated by MPF, respectively. K_a25_ and K_i25_ are the rates of Cdc25 being activated by MPF and inactivated by a phosphatase, respectively. J_a25_ and J_iwee_ are the Michaelis constants of MPF for Cdc25 and Wee1, and J_i25_ and J_awee_ are the Michaelis constants of a phosphatase for Cdc25 and Wee1, respectively.

Investigation of the parameter space through the replica exchange Monte Carlo algorithm (see Methods section ‘Replica exchange Monte Carlo method’) shows that the parameters in our *in silico* model are confined to a small range (Figure S1A) and parameter variations do not change the general trend of the relation between the various rate constants (Figure S1B).

Here we consider each missense mutation as a perturbation to the wild-type status as described in the *in silico* model mentioned above, and the systemic impact of each mutation is projected as the extent that a mutation is likely to deviate from the wild-type state. In our model, entry into mitosis occurs when CycB reaches a concentration (dimensionless) equivalent to an active MPF concentration of 2.0. Assuming cells grow continuously and linearly in time during interphase, the systemic impact of perturbing each rate constant can be gauged through *the change of CycB concentration when active MPF = 2.0*: the higher (lower) the CycB concentration, the longer (shorter) the cell size at mitosis (Figure 1C). Mathematically, this is implemented by calculating the control coefficients C^S^_pi_ that indicate the change of CycB concentration under a consistent amount of perturbation to each rate constant (Methods section ‘Control coefficient’). The sign of C^S^_pi_ shows the direction of CycB concentration change: positive C^S^_pi_ values correspond to an increase of CycB concentration, whereas a negative C^S^_pi_ indicates the opposite. An overall view of C^S^_pi_ calculated for the G2-M mechanism shows that perturbing the Cdc25-related rate constants has a larger impact on the shift of the MPF curve compared to perturbations to the Wee1-related rate constants (Figure S2). This suggests an asymmetric relationship between the positive and negative feedback loops on MPF activation controlled by Cdc25 and Wee1 respectively, which is in agreement with the recent paper by Domingo-Sananes and Novak [18]. Although the unequal impact between Wee1 and Cdc25 has not been confirmed, previous experimental evidence in Xenopus egg extracts [19], [20] suggests this may be the case.

### Mapping missense mutations onto the 3D structures of Cdk1 and CycB

The systemic perturbation of the G2-M transition (Table 2) is studied by examining the effect of four temperature-sensitive mutations in Cdk1 (all mutations except C67Y and G183E) and two temperature-sensitive mutations in CycB that attenuate the function of MPF. The effect of these mutations on protein stability or function is more pronounced when the temperature increases (as proteins are allowed a greater degree of movement). Phenotypically, these mutations allow cells to divide at a greater length than their wild type states when the temperature increases. The cell does divide with the non-temperature sensitive mutant in Cdk1 (C67Y and G183E), but at a smaller cell size.

**Table 2:** *In vivo* length of the yeast trains in the G2-M model.

				Cell Length (µm)		Cell Length (µm)	
Strain Number	Strain Name	Mutated Protein	Residue Change	25°C		30°C	
				Mean	Stdev	Mean	Stdev
275	M35	Cdk1	G43E	16.3	1.5	23.4	6.0
368	3w	Cdk1	C67Y	11.1	1.1	9.8	1.4
8	33	Cdk1	A177T	15.2	1.2	18.2	1.8
154	56/130	Cdk1	G183E	10.4	1.0	12.2	1.9
274	L7	Cdk1	P208S	16.4	1.0	17.6	2.0
515	M63	Cdk1	G227C	16.2	1.3	19.9	2.1
6	NA	CycB	C379Y	14.5	1.4	19.3	2.2
4932	NA	CycB	W395R	18.2	1.0	18.9	1.0
972	WT	NA	NA	12.8	1.6	14.5	1.1

The modeled structure of MPF shows that mutation G43E in Cdk1 is located at the interface of MPF subunits and thus is likely to have a significant effect on the stability of the MPF complex (see Methods section ‘Homology modeling of Cdk1, CycB and MPF structures’ regarding structural modeling). Mutations A177T, G183E and P208S in Cdk1 are located at or close to the active site and hence are likely to cause functional effects; C67Y and G227C in Cdk1 and W395R in CycB are at the periphery of the proteins and thus are mainly structurally related. Mutation C379Y in CycB is within a hydrophobic core and is likely to have a considerable impact on the MPF complex by destabilizing the structure of CycB (Figure 1B).

### SIF values of Cdk1 and CycB mutations correlate to *in vivo* cell lengths

The link between SIF and systemic perturbation (SP) can be statistically established through regression:
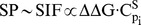
where ΔΔG is the free energy change caused by a mutation to a target protein (here FoldX [21] is applied to approximate the ΔΔG of the mutations studied), which approximates the change in a specific rate constant of the target ODEs (see Text S2 for further information regarding the application of ΔΔG as an evaluation for systemic perturbations); C^S^_pi_ is the control coefficient (Methods section ‘Control coefficient’) that reflects how sensitive the concentration change of the reporter protein (in this case protein CycB) is to the specific parameter.

Hence for the G2-M model the magnitude of each SIF value indicates the degree of impact a mutation can have on the quantity of CycB, which determines when a cell enters mitosis and therefore the length of the cells.

The fundamental concept of our approach is to build a wild-type model that faithfully reflects *in vivo* cellular behavior and then considers each missense mutation as a perturbation to the wild-type status. We do not intend to formulate a model that describes the mutant-type states; we only project the extent that a mutation is likely to deviate from the wild-type state. The procedure of calculating SIF scores is shown in Figure 1. Firstly, a target biological system (in this case the G2-M transition in the cell cycle) is chosen and a reporter protein, whose expression profile can be used to gauge the systemic behavior, is identified. The reporter protein used here is the MPF protein complex. The mutations are then mapped onto three-dimensional protein structures and linked to the associated parameters in the ODEs. For each mutation, its ΔΔG is approximated as the size of perturbation introduced to the associated ODE parameter. To improve the estimation of ΔΔG for each mutation, we applied molecular dynamic (MD) simulations to sample the movement of the flexible regions in the modeled Cdk1 and CycB structures, and then calculated an average ΔΔG based on the sampled conformations (Methods section ‘Molecular dynamic simulations and free energy calculations’). Next, the sensitivity of the expression profile of the reporter protein to each ODE parameter is explored by calculating the C^S^_pi_. Finally, the systemic consequence of each mutation is inferred by calculating its SIF score based on ΔΔG and C^S^_pi_. In the case of the G2-M model, a larger SIF reflects a greater delay for a cell to enter mitosis. Hence a longer cell length should be observed.

**Figure 2:**
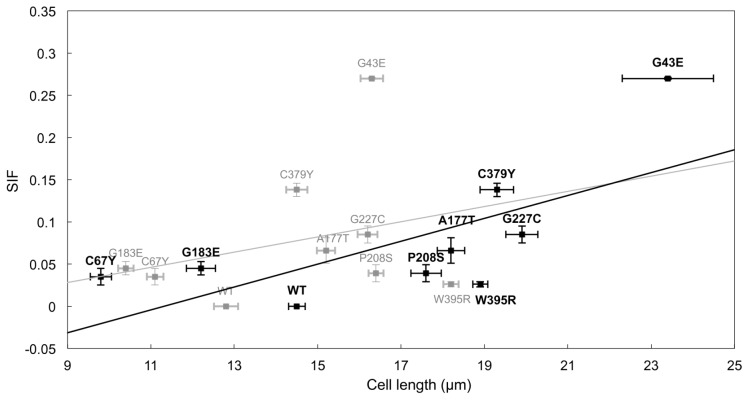
Correlation between SIF and *in vivo* cell length of missense mutations in the G2-M model. The experimentally measured cell lengths and the calculated SIF scores at 25°C and 30°C are shown in grey and black, respectively. The x-axis error bars show the standard error of cell lengths; the y-axis error bars show the standard error of SIF scores, resulting from the evaluation of ΔΔG.

For the eight missense mutation studies presented here, their SIF values are calculated (Table 3) and the length of their host yeast cells are measured at septation (Methods and Material section ‘Yeast strains and cell length measurement’). As shown in Figure 2, the *in silico* SIF score generally reflects the *in vivo* cell length well: at the semi-restrictive temperature (30°C) a medium-to-strong correlation R^2^ = 0.69 (p value = 0.04; all the p values shown in this study are based on the two-tailed model) is obtained.

**Table 3:** *In silico* measurements of the mutant cells in the G2-M model.

Amino acid change	Target Protein	Impact Type^a^	ΔΔG (Cdk1/CycB)^b^	ΔΔG (MPF)^c^	Maximum ΔΔG (kcal/mol)^d^	C^S^_pi_			SIF
						k_d_(CycB)^e^	k_d_ (Cdk1)^f^	J_wee_+J_25_ g	
G43E	Cdk1	S	2.10	24.9^h^	24.9	-	0.011	-	0.27
C67Y	Cdk1	S	3.17	1.31	3.17	-	0.011	-	0.035
A177T	Cdk1	F	5.97	3.65	5.97	-	-	0.011	0.066
G183E	Cdk1	F	3.72	4.13	4.13	-	-	0.011	0.045
P208S	Cdk1	F	3.56	2.35	3.56	-	-	0.011	0.039
G227C	Cdk1	S	7.69	7.23	7.69	-	0.011	-	0.085
C379Y	CycB	S	31.92^h^	34.56^h^	34.56	0.004	-	-	0.138
W395R	CycB	S	6.57	6.15	6.57	0.004	-	-	0.026

a Each mutation is considered to have mainly functional (F) or structural (F) impact according to their locations in its target protein.

b ΔΔG of the mutations in individual Cdk1 or CycB; each of them is an average value considering structures sampled from molecular dynamic simulations.

c ΔΔG of the mutations in Cdk1-CycB complex (MPF); each of them is an average value considering structures sampled from molecular dynamic simulations.

d Maximum of ΔΔG considering both complexed and uncomplexed states of the target protein.

e Perturbation on CycB degradation was weighted 0.3 for the degradation of monomeric CycB and weighted 0.7 for the degradation of complexed CycB (MPF).

f Perturbation on Cdk1 degradation was estimated through the degradation of MPF only since the amount of total Cdk1 is constant.

g Perturbation on the interaction between CycB and Cdk1 was estimated through J_wee_ and J_25_ with a weighting 0.9*J_wee_+0.1*J_25_.

h The high ΔΔG is a result of van der Waals clashes when the target residue is mutated to a larger side chain.

To validate the function of our temperature-sensitive yeast strains, their lengths are also measured at the permissive temperature of 25°C: a condition that allows all the mutants and wild-type cells to grow normally, so the effect of mutation on cell length should be minimal. As shown in Figure 2, there is indeed a much smaller effect of the mutations on cell length at division and a weak correlation (R^2^ = 0.29) between SIFs and *in vivo* cell lengths was observed.

### *In silico* model of the human MAPK pathway

**Figure 3:**
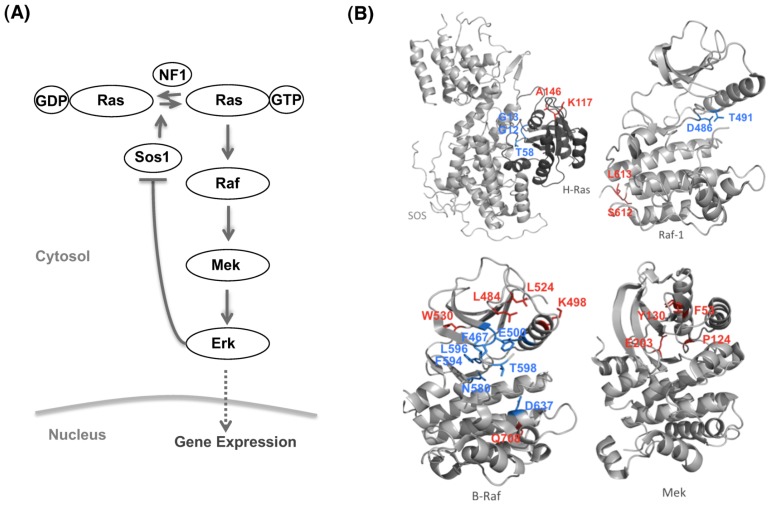
The mutations studied in the MAPK model. (A) A scheme of the MAPK pathway. (B) Mapping the mutations onto the three dimensional structures; mutations located at or close to the active site are colored in blue, otherwise colored in red.

The MAPK pathway plays an essential role in cell survival, proliferation, differentiation and development (Figure 3A). Its three-tier MAPK cascade, i.e. Raf-Mek-Erk, is a highly conserved systemic structure that regulates the switch-like behavior of the pathway's signal transduction mechanism [22]. The important features of this cascade manifest themselves as representatives to evaluate the behavior of the parental pathway, and previous studies of the human MAPK pathway have shown analytical results which support this [23], [24]. To explore the effectiveness of a model that focuses on the dynamics of the three-tier structure, a reduced model is constructed here based on previous work that simulated the signaling cascade from the epidermal growth factor (EGF) receptor to the Erk reporter protein [25]. By omitting redundant terms whose removal has little effect on the expression curve of the downstream protein Erk, a set of succinct ODEs is derived as shown in Table 4 (the derivation is presented in Text S3).

**Table 4:** Differential equations of the reduced MAPK model.

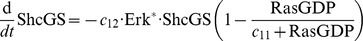
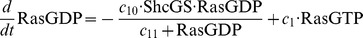
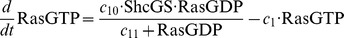
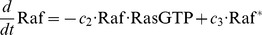
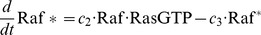
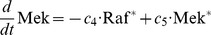
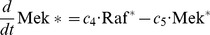
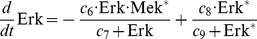
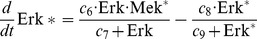
Initial conditions (molecules cell^−1^):
ShcGS = 20,000; RasGDP = 20,000; RasGTP = 0; Raf = 10,000;
Raf* = 0; Mek = 360,000; Mek^*^ = 0; Erk = 750,000; Erk^*^ = 0
Rate constants (molecules^−1^ cell min^−1^):
c_2_ = 7.7⋅10^−4^; c_6_ = 8.3; c_8_ = 4⋅10^5^; c_10_ = 15; c_12_ = 4⋅10^−6^
Rate constants (molecules cell^−1^):
c_7_ = 9⋅10^4^; c_9_ = 6⋅10^5^; c_11_ = 1.53⋅10^3^
Rate constants (min^−1^):
c_1_ = 69; c_3_ = 14; c_4_ = 50; c_5_ = 0.78

c_1_ and c_2_ are the rate and Michaelis constant for RasGDP activation by the Shc-Grb-Sos (ShcGS) complex, respectively; c_3_ is the rate for RasGTP to be converted to RasGDP; c_4_ is the rate for RasGTP to convert Raf from an inactive to an active form (Raf^*^); c_5_ is the rate for RasGTP to convert Raf^*^ to Raf; c_6_ is the rate for Raf^*^ to convert Mek from an inactive to an active form (Mek^*^); c_7_ is the rate for Mek^*^ to be converted to Mek; c_8_ and c_9_ are the rate and Michaelis constant, respectively, for Mek^*^ to convert Erk from an inactive form to an active form (Erk^*^); c_10_ and c_11_ are the rate and Michaelis constants, respectively, for Erk^*^ to be converted to Erk. Finally, c_12_ is the rate for the ShcGS complex to be inhibited by Erk^*^ (See simulated curves in Figure S3).

**Figure 4:**
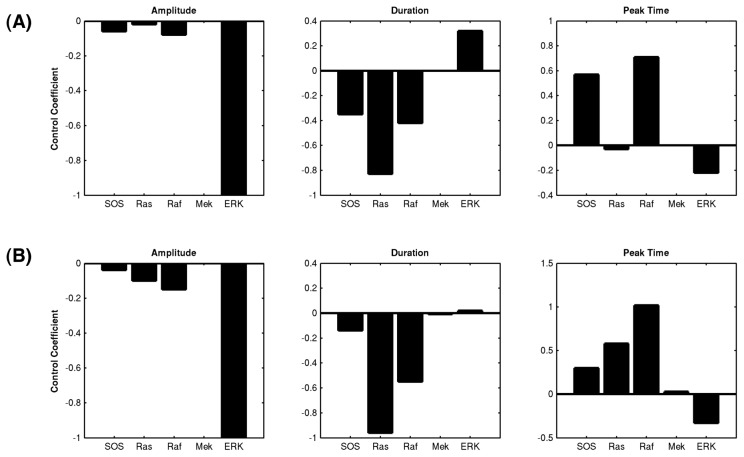
The sensitivity of the key proteins in the MAPK pathway in terms of regulating the Erk expression. (A) The reduced G2-M model. (B) The original non-reduced (Brightman and Fell) model.

To benchmark the behavior of both the reduced and original model, a sensitivity analysis is performed over three target quantities of the reporter protein Erk (Methods section ‘Quantifying the change of expression curves’): the amplitude (maximum activation), duration (time until signal drops down to 50% of its maximum activation) and peak time (time of maximum activation). For the test, the initial concentration of the key proteins in both models is varied and their effects on controlling the target quantities of Erk is compared (the key proteins include ShcGS (Shc: Src homology and collagen domain protein), GS, Grb2 (growth factor receptor binding protein 2), SOS (son of sevenless homologous protein), Ras, Raf, Mek and Erk). As a result, the control coefficients in both models demonstrate a similar pattern across all three-target quantities (Figure 4A,B), which indicate that the reduced model does not sacrifice the overall dynamics of the original model to achieve its simpler structure.

### Mapping missense mutations onto the 3D structures of H-Ras, Raf-1, B-Raf and Mek

Here 40 mutations associated with neuro-cardio-facial-cutaneous syndrome are collected and studied (Table S1). As shown in Figure 3B, all the mutations can be mapped to crystal structures of H-Ras, Raf-1, B-Raf and Mek, and each mutation is classified as mainly functionally or mainly structurally important according to its location in the target protein.

### H-Ras mutations cause different systemic effect from other mutations

**Figure 5:**
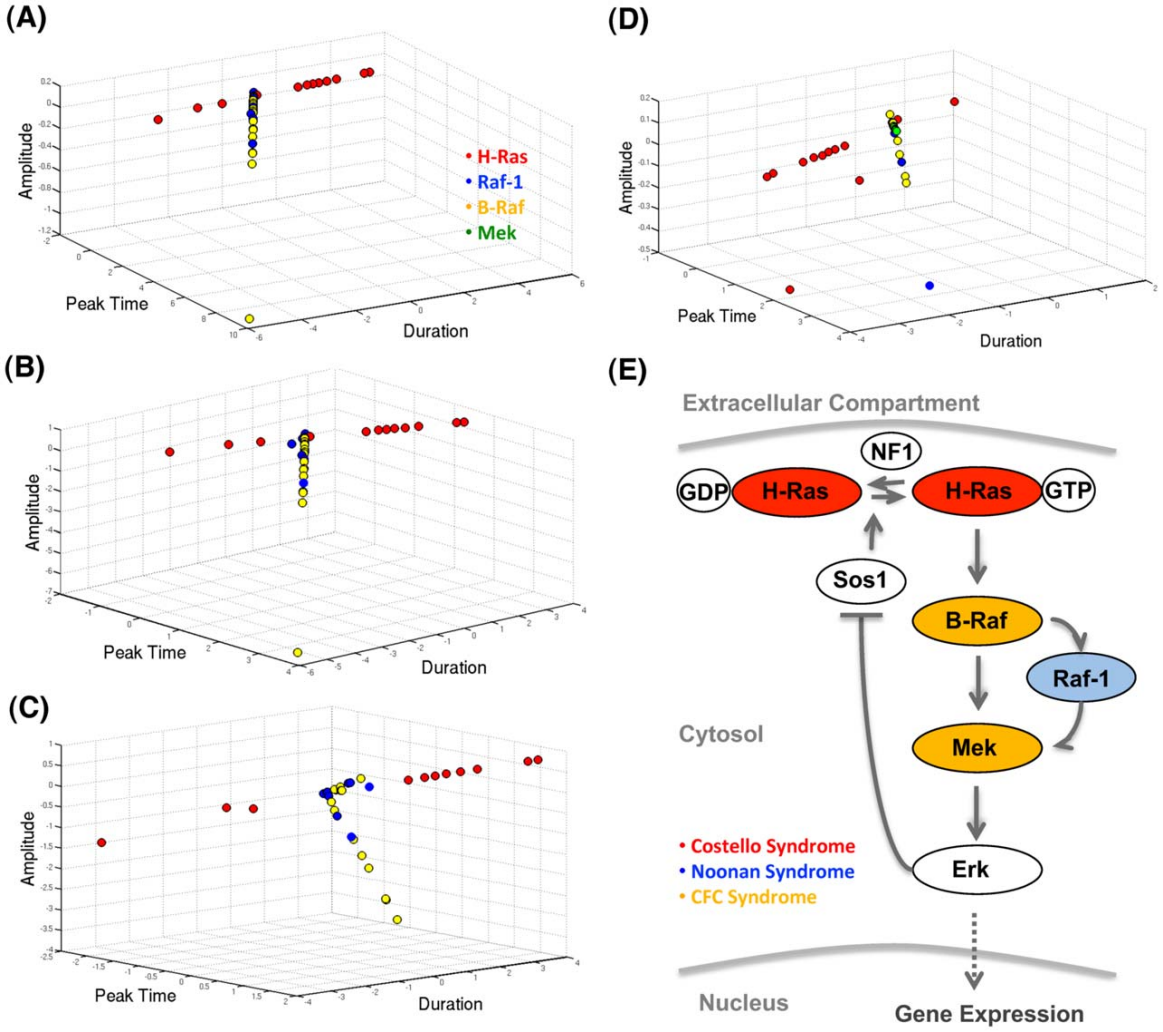
(A)–(D) shows the SIF cores of the mutations studied in the MAPK model. (A) The reduced model; (B) the reduced model with initial conditions from Fujioka et al; (C) the reduced model with initial conditions from Fujioka et al and parameters optimized by fitting to the time course data in Fujioka et al; (D) the original non-reduced model. (E) A scheme shows the relationship between the key proteins and their clinical syndromes.

Unlike missense mutations in the yeast G2-M model, there are no quantitative measurements of the physiological outcomes for the mutations in the MAPK pathway that can be used to calculate the correlation with SIF scores. Hence, as an indirect way to evaluate the relationship between mutations and clinical symptoms, each mutation is represented by three SIF scores calculated according to the systemic impact on the wild-type Erk expression curve: measured as amplitude, duration and peak time differences. The trajectory of the SIFs corresponding to each mutation as a function of these three target quantities shows that mutations in Raf1, B-Raf and Mek are more likely to be overlapped in a similar region, whereas mutations in H-Ras tend to distribute in a very different trajectory to the direction of the other mutations (Figure 5A). To determine if the different distribution of H-Ras mutations is a robust feature, a different set of initial concentrations that were measured experimentally in HeLa cells by Fujioka et al. [26] is used to derive two new parameter sets: one produces expression curves similar to those of the original model, whilst the other one produces curves fitted to the *in vivo* FRET data measured by Fujioka et al [26] (the parameters of both models are available in Text S1). As shown in Figure 5B and 5C, both parameter sets distribute H-Ras mutations in a trajectory different from other mutations, which suggests that the separation of H-Ras is not sensitive to variations to initial concentrations and parameter space. As a benchmark, the three dimensional SIF scores from the original model are also presented (Figure 5D). Consistently, H-Ras mutations are distributed into a distinctly different group.

It has been demonstrated that using ensembles of simulated protein structures, rather than a single conformation as represented by a crystal or modeled structure, can improve the estimation of free energy change [27]. In order to determine if the use of structural ensembles affects the SIF distribution, molecular dynamic (MD) simulations are also applied to sample the movement of flexible regions in the key kinases. Eventually an average ΔΔG, and therefore an average SIF score, was calculated for each mutation based on the alternative structures sampled by the MD simulations (Methods section ‘Molecular dynamic simulations and free energy calculations’). By using the average SIF scores calculated over the conformation ensemble, a less narrow distribution for B-Raf and Raf-1 mutations is observed in the reduced model with parameters fitted to the experimental FRET data while the distribution of mutations in other models remained largely unchanged (Figure S4). Moreover, the overall distribution of the SIF scores in all of the models is in agreement with the results using only the crystal structures. This suggests that the SIF scores are not overly sensitive to movements away from the experimentally determined atomic positions.

A closer examination of mutant SIF scores reveals that H-Ras mutations perturb the MAPK pathway in a distinctly different manner from that of the mutations in Raf-1, B-Raf and Mek (Figure 5A–D): H-Ras mutations tend to dominantly affect the duration of the Erk expression profile whereas the other mutations mainly affect the amplitude of the expression profile, followed by a smaller impact on peak time and an even smaller effect on the duration of the Erk activation. Physiologically, this indicates that the cellular response to H-Ras mutations is different to the other mutations. Indeed, the duration of Erk activation is known to be a critical factor for determining cell fate: in PC12 cells, it has been shown experimentally that prolonged activation of Erk is sufficient for cell differentiation whereas transient activation is associated more closely with cell proliferation; in fibroblast, a reverse relationship between duration and cell fate is observed [28]. Although the amplitude of Erk activation has also been experimentally shown to be a determinant of cell fate, its effect is more complicated: high level of Erk activation usually promotes cell-cycle progression but sometimes it leads to cell-cycle arrest as well [28]. Also, the mutations in Raf-1, B-Raf and Mek mainly reduce the amplitude of Erk expression and hence it is likely that they have less effect on cell growth than H-Ras mutations, which mainly increase the duration of Erk expression.

## Discussion

### Systemic impact is a result of protein stability change and pathway perturbation

In this work we presented the SIF function as an effective measure for the systemic impact of missense mutations. SIF values reflect in a simple manner the fact that proteins are functional units in the cell whose interaction networks regulate cellular behavior. It is of particular interest to see that SIF scores reflect the *in vivo* phenotype in the yeast G2-M model when there is no additional parameter introduced to distinguish functionally and structurally important mutations. This suggests that, although they change protein behavior in different ways, functional and structural mutations can perturb a pathway to a similar extent.

A potential way to improve the current correlation between SIF and systemic outcome is to consider an additional parameter λ that describes the amount of parameter perturbation caused by free energy change. Now the SIF function becomes:
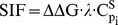
By assigning different λ constants for functional and structural mutations in the G2-M model, we found that using a larger λ for functional mutations consistently provides smaller correlations (less than 0.68). This suggests that ΔΔG in this case over-estimates the systemic impact of functional mutations and thus should be scaled down by a smaller λ when used for analyzing mutations at functional sites. This also indicates that functional mutations may be better annotated by considering other protein-protein interactions besides protein stability. However, this would make it much more difficult to quantify the impact of protein interactions. Using a smaller λ for functional mutations may be suitable for the mutations studied here; nevertheless further investigation is required to determine if this is a general criterion that could be applied for mutations in other biological systems.

When we considered only structural mutations in the G2-M model, the correlation between SIF and cell length increases from 0.69 to 0.73 (p value = 0.026). This suggests that the current SIF formula may perform much better in annotating the systemic effect of mutations whose role is more structural than functional. This could be due to the way we approximate the functional impact of a missense mutation through Michaelis constants and link its perturbation to ΔΔG as an approximation of K_d_ (Text S2).

Although the current SIF function correlates linearly with *in vivo* measurements, the data cannot rule out an exponential relationship between SIF and phenotypic outcome. As described in Text S2, if we broadly approximate the amount of perturbation in each rate constant to be 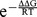, SIF can be formulated as 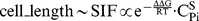 ∼To approximate the direct use of ΔΔG, we may transform the SIF function to 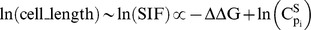. Following this formula, the correlation between ln(cell length) and ln(SIF) is reasonable: 0.62. Further studies will be required to explore the optimal correlation between SIF and systemic effects.

### Simple models are beneficial for extrapolating systemic impacts

A very intriguing result of this study is that systemic impacts can be reasonably gauged through simple or reduced ODEs. This indicates that it is possible to study the systemic perturbation of a pathway when there is incomplete information about its components – an important observation, given the fact that the majority of biological pathways have missing components waiting to be discovered or confirmed. Another import aspect of this work is that, for the purpose of studying systemic perturbation, it is feasible to study the missense mutations through “fuzzy” parameters – that is, the systemic impact of a mutation can be extrapolated through rate constants that account for general protein-protein interactions rather than detailed enzyme catalytic reactions. Finally, the advantage of using a simpler model is also reflected in facilitating a lower chance of associating multiple parameters with a perturbation, which means the difficulty of discussing the impact of a missense mutation can be reduced.

The simplicity of the G2-M model lies in two aspects. First, it has only four major component proteins (Cdk1, CycB, Wee1 and Cdc25) used to simulate cell growth, and the model can be considered to be linear, terminating when MPF reaches a certain critical concentration. The second aspect is that, rather than capturing their time-course data, the model reflects the relationship between the component proteins. Normally this raises the difficulty of parameter optimization, as it increases the chance of converging to multiple parameter sets that all give simulation curves satisfying a particular phenotypic outcome. Fortunately, parameter inference is not a concern in this case, since the general trend of the C^s^_pi_ relation between parameters is conserved, regardless of parameter variations (Figure S1B), i.e. the correlation between the SIF values and *in vivo* cell lengths is not sensitive to parameter variation.

In preserving the overall dynamics of the original model (Figure S5), our reduced MAPK model is efficient in terms of parameterization; it has only 12 rate constants, compared to the original 27. The simplified ODEs allow us to conduct a straightforward analysis on missense mutations, which may not be the case in a more complicated model. For example, a mutation in the functional site of Ras can affect two downstream interactions in the original model (see Figure S5: one is between Ras and Gap; the other is between Ras and Raf), whereas it can only affect the interaction between Ras and Raf in the reduced model. Furthermore, without reduction we would not be able to implement the robustness test on the SIF projections shown in Figure 5, since it is most unlikely that one could obtain robust parameters given the expression data profile from Fujioka et al [26]. The practicality of a simpler model suggest that the idea of using ODEs to model the dynamics of a pathway can be more feasible than previously thought, as long as we can reduce a complex pathway to smaller modules that account for the functional core of a pathway.

### Investigating systemic perturbation helps to understand the phenotype or underlying mechanism of missense mutations

It is generally non-trivial to infer cellular phenotypes from studying pathway dynamics since many cellular functions have complex underlying mechanisms. However, the medium-to-strong correlation between the SIF values and *in vivo* yeast cell lengths in the G2-M model shows that it is possible to estimate effectively the phenotypic effect of missense mutations through gauging systemic impacts. This is due to two essential factors underlying our G2-M model. Firstly, cell length at septation (cell division) is a faithful indicator for identifying cells at the beginning of mitosis. This is because fission yeast grows only in length and thus it can be positioned in its cell cycle simply by its length and does not grow between entry into mitosis and septation. Secondly, the chosen reporter protein, MPF, is closely linked to the initiation of mitosis. A strong support for this is a recent discovery that MPF is a necessary and non-redundant factor for triggering mitosis [29].

The SIF values simulated from the MAPK model, on the other hand, reflect a more complex relationship with phenotype. We expected that most of the mutations studied here should be projected into similar regions, as they are associated with overlapping symptoms under a broad term ‘neuro-cardio-facial-cutaneous syndrome’. However, H-Ras mutations are projected into distinctly different trajectories from the other mutations with respect to their effects on the ERK expression profile. This suggests that H-Ras mutations are likely to have different characters in terms of the disease prognosis and risk of complications depending more upon the genotype than on the phenotype. Given the clinical symptoms of patients from which the missense mutations studied here were identified (as shown in Figure 5E, all the H-Ras mutations are associated with Costello syndrome (CS); most of the Raf-1 mutations are associated with Noonan syndrome (NS); most of the B-Raf and all of the Mek mutations are associated with cardio-facio-cutaneous syndrome (CFCS)), the result in Figure 5A–5D suggests that NS and CFCS may share some degree of similarity in terms of disease development. Indeed, it is often difficult to distinguish an infant with CFCS from NS, although the phenotype becomes more distinctive with time [30]. Interestingly, current knowledge of the genotype-phenotype correlations suggests that the presence of mutations in the H-Ras gene is associated with a much higher tendency of cancer compared to the other mutations [31], indicating a potentially different system dynamic, as indeed demonstrated in this study. As a whole, the MAPK model serves as a good example to show how qualitative annotation of mutations (the classification of mutations) can contribute to the understanding of disease mechanisms. This is practically useful as it is often hard to clinically quantify various disease phenotypes that lead to differences in prognosis and drug response.

The two systems in our study show that SIF can reflect phenotype or the underlying mechanism of missense mutations in proteins. In general, we may confidently interpret systemic impacts as an indicator for phenotype only if a reporter protein is strongly and non-redundantly linked to a target phenotype; otherwise a more reserved view would be appropriate.

### Potential limitations of SIF

One confounding factor associated with the performance of SIF is the relationship between ΔΔG of a mutation and its actual phenotypic effect. This is because different proteins may have different stability states and hence they may respond differently to the same amount of ΔΔG caused by missense mutations. The issue of benchmarking the effect of ΔΔG on different proteins has been an active topic in annotating nsSNPs. Previous studies show that proteins belonging to different structural families can respond differently to the same amount of ΔΔG, but in general a small margin of ΔΔG (1–3 kcal/mol) can be approximately used to define missense mutations that may not cause an immediate effect on protein fitness [32], [33], [34]. On the other hand, for proteins that share Immunoglobulin-like folds, a clearer phenotypic threshold of ΔΔG (2 kcal/mol) can be used to define missense mutations that generally result in severe disease phenotypes [35], [36]. Hence, taking a more stringent view, this implies that proteins sharing similar structures are more likely to react similarly to mutations that cause the same amount of ΔΔG.

For the proteins studied in this work, the concern of comparing the effect of ΔΔG across different proteins is likely to be alleviated due to the above reasoning. In the G2-M model, CycB and Cdk1 form a complex and hence the uncertainty of comparing ΔΔG in two different proteins is reduced. In the MAPK model, all the key proteins are kinases that share the same well-structured fold.

Another factor that may affect the performance of SIF is the complication of assigning the role of a mutation as mainly functional or structural. This issue is especially hard to deal with if a missense mutation is likely to cause long-range structural effects on its host proteins - for example, a mutation can exist far away from a functional site (and thus is considered as a structural mutation) but still affect the function of its host protein by inducing long-range conformational changes. Hence additional attention should be paid to calculating SIF for mutations located in proteins that are not well studied or have versatile conformations. For the cases studied in this work, the problem of assigning functional and structural mutations is not significant because most of the key proteins are kinases that have well-defined functional sites (see Text S2 for further information).

One other factor that is associated with SIF performance is the accuracy of calculating ΔΔG. So far most of the methods for predicting ΔΔG do not show a good correlation with the experimental ΔΔG; however, they do perform well when used to estimate the average effect of mutations on protein stability [37]. This is likely to support the good correlation between SIFs and the *in vivo* cellular phenotypic outcomes measured in our study, since we calculated an average ΔΔG for each missense mutation based on the simulated structures and used it to correlate with the experimental data.

Finally, it is worth mentioning that the performance of SIF can be considerably compromised by mutations with large ΔΔG values. These mutations can be too extreme to be considered a perturbation to a target system, and hence the ODE model describing the wild-type condition is not applicable. On the other hand, large ΔΔG values can also be the result of Van der Waals clash that are often heavily penalized in ΔΔG calculations (as likely the case for mutations G43E and C379Y in the yeast G2-M model). All in all, in the cases where ΔΔG is large, caution should be taken when applying the SIF function.

### Concluding remarks

Our study as a whole suggests that it is beneficial to combine multi-level knowledge to investigate the effects of missense mutations on cellular behavior. The advance in protein structure prediction techniques will particularly make the calculation of SIF more feasible, since it requires the structural information of proteins that host the target missense mutations. Overall, there is sufficient reason for us to be confident that future studies on integrating protein and pathway dynamics will become increasingly viable, as there are constant efforts across the scientific community in solving protein structures and identifying new components in biological pathways.

Simulating pathway dynamics through ODEs, as demonstrated here, provides a convenient platform for utilizing the information on protein structures. However, the application of ODEs implies two major limitations. One is in the availability of time-course data of protein expression in public resources; at the moment this is relatively low and sparse compared to that of gene expression data. This will be alleviated as more high-throughput time course data becomes available. The other limit is in our knowledge of the biological pathways – a majority of them have only been partially uncovered. A feasible way to circumvent the problem is to develop a simpler model by considering only key proteins that are essential for preserving pathway behaviors, as we have demonstrated in the case of MAPK pathway and G2-M transition.

The SIF function in its current form gives a good approximation of systemic perturbation resulting from the missense mutations in the G2-M and MAPK models. With further development on a larger dataset, especially with the inclusion of more parameters to further characterize protein function and structure, we are likely to obtain better correlations with quantitative phenotypes. The process of refining the SIF equation will tell us more about the relationships between protein function and structure, and pathway dynamics, which is one of the most important questions considered by structural biologists.

The advance of high throughput technology has enabled us to identify mutations in a large number of inter-connected pathways. It is becoming apparent that performing experiments to check the impact of individual mutations on the pathway level will be extremely time-consuming and costly, let alone monitoring all the possible cross-interactions and combinatorial effect of multiple mutations. From this perspective, multi-level mathematical modeling, such as that described here, will provide an efficient mechanism for pre-screening systemic impact in a cost-effective way. This is particularly useful for studying the etiology of complex diseases that are usually the result of accumulating multiple mutations.

## Materials and Methods

### Yeast strains and cell length measurement

Yeast strains used in this study are listed in Table 3.

All the strains except strain 4932 were generated following our protocol previously published by Nurse et al. [38]. Strain 4932 was generated and identified as described in the work of Fong et al. [39] with the following changes: Genomic DNA from a cdc13hph tagged strain was used as the starting template. TaKaRa LA-Taq polymerase (Takara Bio) was used for the first round of PCR and Z-Taq (Takara Bio) for the mutagenic PCR reaction that was supplemented with 10XdGTP. Mutation positions were identified using Big Dye (Applied Biosystems) terminator cycle sequencing.

Cells were grown to mid exponential growth (∼5×10^6^ cells/ml) in rich media at 25°C and 30°C [40] and photographed using a Zeiss Axioplan microscope. Cell lengths upon mitosis, by unbiased sampling of 30 septated cells, were measured using ImageJ.

### Replica exchange Monte Carlo method

Here we applied the replica exchange Monte Carlo method (REM) – also known as parallel tempting (PT) – to implement parameter inference. For a non-linear system, as represented by the G2-M and MAPK model, the energy surface is normally rugged and it is hard to ensure unbiased sampling along the uneven energy space. Nevertheless REM has been shown to be very useful for this purpose, especially at low temperatures, and has been used extensively for finite-temperature simulation of biomolecules [41], [42]. The general idea of REM is to simulate a number of subsystems {X_(m)_} with different inverse temperatures β_m_ (*replicas*) in parallel. At particular intervals, the sampling trajectory is exchanged from one subsystem to the others (usually adjacent replicas) with the following probability specified in [43]:
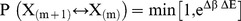
where Δβ = β_m_+1−β_m_ is the difference between the inverse temperature of the two replicas and ΔE = E (X_(m+1)_)−E (X_(m)_) is the energy difference between them (in our case the deviation of the protein expression time course).

Practically, the exchange of replicas with different temperatures effectively generates repeated heating and annealing cycles, which avoids the parameter search from becoming trapped in a local energy minimum.

For sampling the trajectories, PEPP used the Metropolis algorithm [44] with modifications that allow uneven sizes of sampling steps. This echoes the idea that the coexistence of large and small changes in phase space is essential for sampling unstable structures [45]. To determine the size of each sampling step (Δx), PEPP adopted the method introduced in [45]:
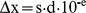
where d and e are random integers uniformly distributed in d
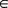
[1,9] and e
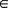
[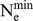, 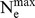] and s is a binary random number that is either 1 or −1 with probability of 0.5. 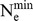 and 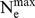 determine the logarithmic scales of the smallest and largest step move, respectively. As a result, the overall sampling density is a mixture of uniform distributions with different scales; it has a sharp peak near zero and very long tails.

The iteration of the Metropolis algorithm in our model is as following:

1. Introduce a perturbation (Δx), whose scale is determined according to the last function shown above, to the initial parameter (X) in a target system.
2. Run the simulation with the initial X and the perturbed X′, which generates the respective energy E and E′.
3. Draw a uniform randomly number R
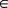
[0,1]. Whether X′ is accepted as the next move of sampling depends on 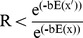:
4. Return to step 1)

### Quantifying the systemic impacts of the mutations

#### Control coefficient (C^S^_pi_)



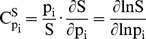
where p_i_ is the parameters in the model and S is a downstream reporter that is tightly associated with a specific phenotype.

In both G2-M and MAPK models, was calculated given ∂p_i_ = 0.1. For mutations that can be associated with two rate constants, e.g. X and Y, ∂p_i_ is defined as ∂p_i_^X^+∂p_i_^Y^ = 0.1.

#### Quantifying the change of expression curves

As mentioned above, is calculated based on ∂S that is the deviation between wild type and mutant type curves of a reporter protein. In the G2-M model, the deviation of the CycB curve is measured as the concentration change of CycB when MPF reaches a dimensionless concentration 2.0. In the MAPK model, the deviation of the Erk curve is measured in three dimensions that are commonly investigated in studying pathway behavior: (1) peak difference, i.e. the difference of the maximum activation; (2) duration difference, i.e. the difference of time until the signal drops down to 50% of its maximum activation; (3) peak time difference, i.e. the difference of the time that the curves reach its maximum activation (Figure S6).

### Structural analysis of the target proteins

#### Homology modeling of Cdk1, CycB and MPF structures

We modeled Cdk1 using human cyclin-dependent kinase 2 (CDK2, PDB code: 1FIN, chain A, sequence identity shared with Cdk1: 64%) as a template. Here we employed MODELLER (version 9v2; set deviation = 4.0; number of models = 50; call routine = ‘model’) [46] to construct the three-dimensional structure of Cdk1, guided by a sequence to structure alignment between the query and the template using the program JOY [47] (Figure S7A). Examination of the model by PROCHECK [48] shows 90% of the Cdk1 model's backbone angles are within the core region of Ramachandran plot while only one loop residue (L37) is within the generally allowable region (Figure S7B). For the case of CycB, it was modeled by applying JOY and MODELLER using both human Cyclin A (PDB: 3DOG, chain B, sequence identity shared with CycB: 36%) and human cyclin B (PDB: 2JGZ, chain B, sequence identity: 40%) as templates (Figure S8A). Evaluation of the model by PROCHECK shows that 93% of the modeled CycB backbone angles are within the core region and only one loop residue W249 is within the generally allowable region (Figure S8B). For all constructed models, there are no backbone angles, other than for the residue glycine, in disallowed regions of the Ramachandran plot. Lastly, the MPF complex was modeled by superimposing modeled Cdk1 and CycB onto the human Cdk2-Cyclin A complex (1PDB: 1FIN).

#### Molecular dynamic simulations and free energy calculations

AMBER10 is employed with the ff99SB force field [49], [50] to generate conformation ensembles for each mutation studied. Each target protein is solvated by water molecules. For proteins with surface charges, salt ions are included in the solution for maintaining stable protein structures. Each protein is first equilibrated by a 50 picoseconds heating phase to 300 K, and 50 picoseconds of density equilibration with weak restraints on the structure. This is followed by a simulation at constant pressure at 310 K (300 K for the proteins in the yeast system) up to 100 picoseconds. All simulations are run with the shake algorithm applied to hydrogen atoms, a 2 femtoseconds time step and Langevin dynamics for temperature control. For the final phase of equilibration and subsequent simulation up to 100 nanoseconds, the following parameters were used: imin = 0, irest = 1, ntx = 5, nstlim = 50000000, dt = 0.002, ntc = 2, ntf = 2, cut = 8.0, ntb = 2, ntp = 1, taup = 2.0, ntpr = 50000, ntwx = 50000, ntt = 3, gamma_ln = 2.0, temp0 = 310 (300 for the yeast system).

For each mutation, 100 simulated structures are sampled across the total 100 nanoseconds simulation time. The average ΔΔG is then calculated based on the Boltzmann-Gibbs distribution as discussed in [51]: 
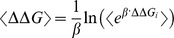
, where 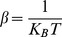, K_B_ is the Boltzmann constant and T is temperature, and ΔΔG_i_ is individual sampled structure. Finally, an average SIF score for each mutation is calculated based on 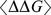.

FoldX (version 3.0) is employed to calculate the ΔΔG for each mutation. Prior to the calculation of ΔΔG, the RepairPDB command in FoldX is used on each sampled structure to fix non-standard angles, distances and side-chain conformations. The default setting of FoldX is used to calculate the ΔΔG of each mutation: Temperature = 298 K, pH = 7, IonStrength = 0.050, VdWDesign = 2.

## Supporting Information

Figure S1 Checking the robustness of parameters in the G2-M model. (A) Error distribution of the parameter sets sampled 1,000 times with random starting points shows two major clusters of parameters. The first cluster of parameters have chi-squared errors <5 and the other have chi-squared errors between 5 to15 (the error estimates the difference between the simulated curves and experimentally observed ones). The cluster with smaller errors produce curves similar to those from the original Novak and Tyson (1993) model whereas the other with larger errors results in a flat curve of CycB and MPF. Therefore, in this case only the parameters with error <5 are considered. (B) Control coefficients (C^S^_pi_) of parameter sets that are close to local minimum of parameter inference, i.e. within the cluster that have smaller errors.
(PDF)

Figure S2 Asymmetric control of Wee1 and Cdc25 on the G2-M model. The absolute values of the control coefficients for Cdc25-associated reactions are larger than those for Wee1.
(PDF)

Figure S3 Simulated curves for the MAPK model. (A) The reduced model (solid lines) and the original Brightman and Fell model (dashed lines). (B) The reduced model with initial concentrations measured by Fujioka *et al.* (solid lines) and the expression data from Fujioka *et al.* (dashed lines). (C) The reduced model with initial concentrations measured by Fujioka *et al.* plus parameter sets optimized according to the FRET data measured by Fujioka *et al.* (solid lines) and the expression data from Fujioka et al [26].
(PDF)

Figure S4 The SIF scores of the mutations in the MAPK model considering conformational ensembles. (A) The reduced model; (B) the reduced model with initial conditions from Fujioka *et al.*; (C) the reduced model with initial conditions from Fujioka *et al.* and parameters optimized by fitting to the time course data in Fujioka *et al.*; (D) the original non-reduced model.
(PDF)

Figure S5 An overall structure of the original and reduced model. (A) The original non-reduced model and (B) the reduced model.
(PDF)

Figure S6 The three measurements used to quantify the difference between two proteins expression curves.
(PDF)

Figure S7 Structural analysis of Cdk1 model. (A) The alignment of Cdk1 sequence and the template structure PDB: 1FIN. The structure features of the template are shown in the JOY [52] format: each alpha helix is indicated in red, beta strand in blue and 3_10_ helix in maroon. Solvent accessible residues are shown in lower case, solvent inaccessible in upper cases. Residues hydrogen bonded to main-chain amide groups are shown in bold style; those hydrogen bonded to main-chain carbonyl groups are underlined. Positive phi torsion angle in italic style; disulfide bonds are indicated with cedilla (B) Ramachandran plot of the Cdk1 modeled structure. Residues that have a less favorable but generally acceptable backbone conformation are highlighted in red.
(PDF)

Figure S8 Structural analysis of CycB model. (A) The alignment of CycB sequence and the template structures PDB: 2JGZ and 3DOG. The structural features of the template are shown in the JOY [52] format, as explained in the legend of Figure S7. (B) Ramachandran plot of the CycB modeled structure. Residues that have less favorable but generally acceptable backbone conformation are highlighted in red.
(PDF)

Figure S9 Simulated curves of the reduced and original models. (A) Comparison of the relative activation of Mek, for the Brightman and Fell model (solid lines), and the situation where the Mek activation is replaced by Eqn. 21 (dashed line). Here note [Mek*] = [MekP]+[MekPP]. (B) Comparison of the relative activation (concentration of active form, divided by initial concentration of protein) of ErkPP between the original Brightman and Fell model (solid line) and the simplified version in which the Erk activation is replaced by Eqn. 24 (dashed line). (C) Comparison of the relative activation between the Brightman and Fell (2000) model (heavy lines) and the equivalent simplified model (light lines).
(PDF)

Figure S10 Three-dimensional structure of a kinase. The G-rich loop is colored in green; the C-alpha helix is colored in magenta; the catalytic loop is colored in orange; the activation loop is colored in cyan. The N-lobe region is colored in black while the C-lobe is colored in grey.
(PDF)

Table S1 Mutations and model parameters associated with neuro-cardio-facial-cutaneous syndrome.
(DOC)

Text S1 Additional information of the *in silico* MAPK model. This file describes the parameter values optimized based on the FRET data measured by Fujioka et al.
(DOC)

Text S2 Characterizing structural and functional mutations. This file discusses the separation of structural and functional mutations studied in this work.
(DOC)

Text S3 Formulating the reduced ODEs based on the Brightman and Fell model. This file describes the steps of producing the reduced model.
(DOC)
