# Supplementary figures and images for: A Structural Systems Biology Approach for Quantifying the Systemic Consequences of Missense Mutations in Proteins
Source: PLoS Comput Biol. 2012 Oct 18;8(10):e1002738. doi: 10.1371/journal.pcbi.1002738 (PMC3475653; doi:10.1371/journal.pcbi.1002738)

Figure S1

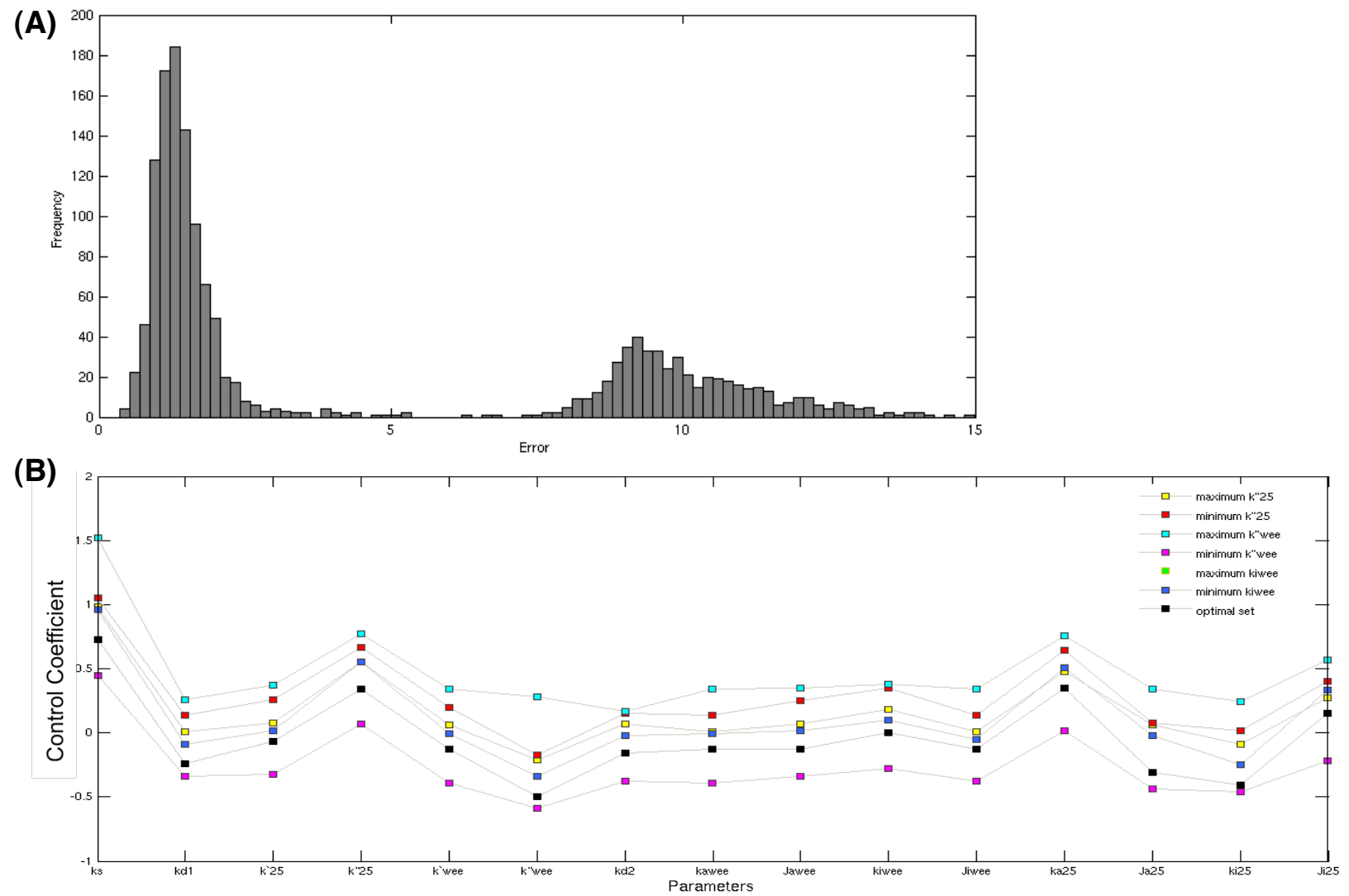

Supplement: Figure S1 — Checking the robustness of parameters in the G2-M model. (A) Error distribution of the parameter sets sampled 1,000 times with random starting points shows two major clusters of parameters. The first cluster of parameters have chi-squared errors <5 and the other have chi-squared errors between 5 to15 (the error estimates the difference between the simulated curves and experimentally observed ones). The cluster with smaller errors produce curves similar to those from the original Novak and Tyson (1993) model whereas the other with larger errors results in a flat curve of CycB and MPF. Therefore, in this case only the parameters with error <5 are considered. (B) Control coefficients (CS pi) of parameter sets that are close to local minimum of parameter inference, i.e. within the cluster that have smaller errors. (PDF) [file pcbi.1002738.s001.pdf]

Figure S2

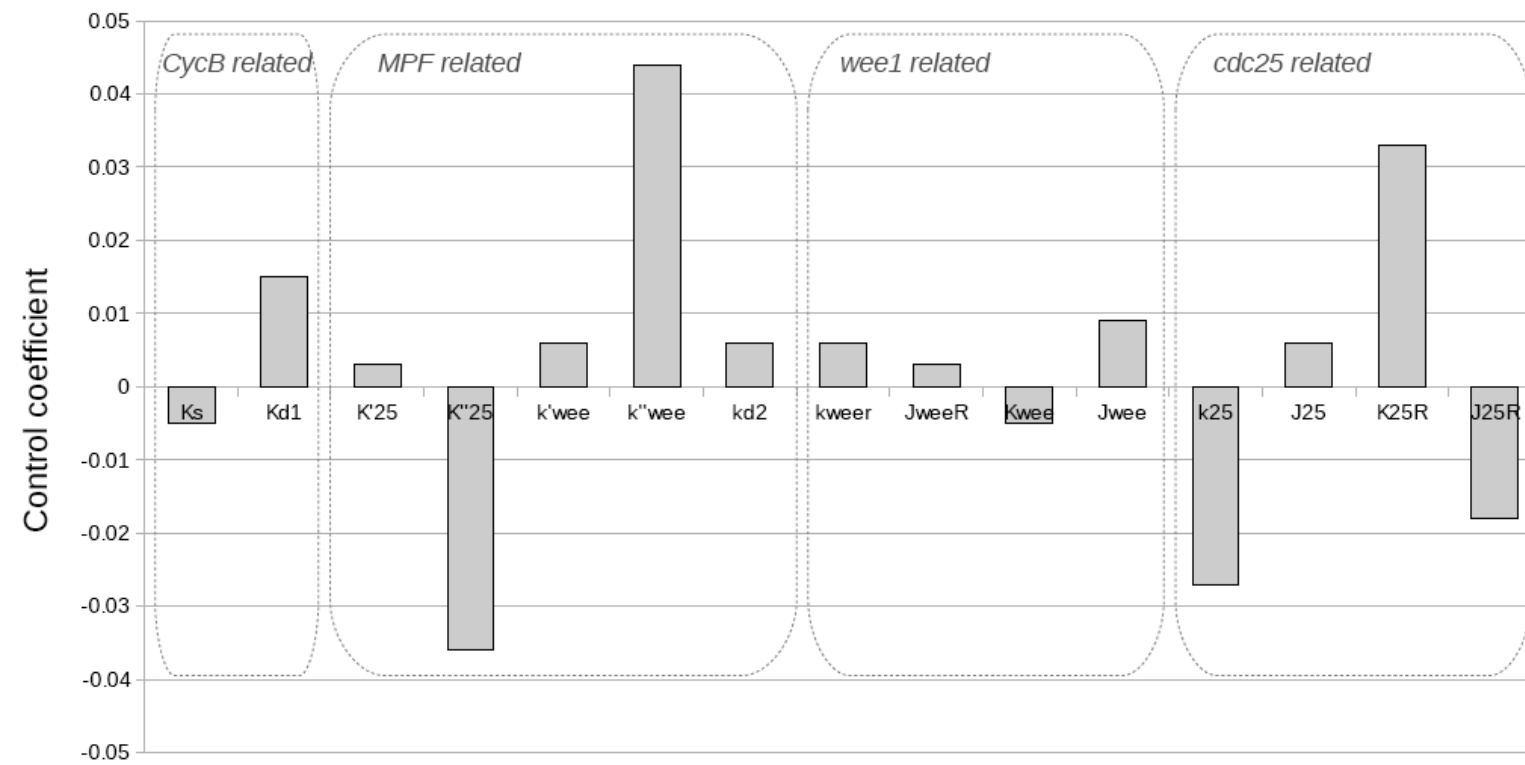

Supplement: Figure S2 — Asymmetric control of Wee1 and Cdc25 on the G2-M model. The absolute values of the control coefficients for Cdc25-associated reactions are larger than those for Wee1. (PDF) [file pcbi.1002738.s002.pdf]

Figure S3

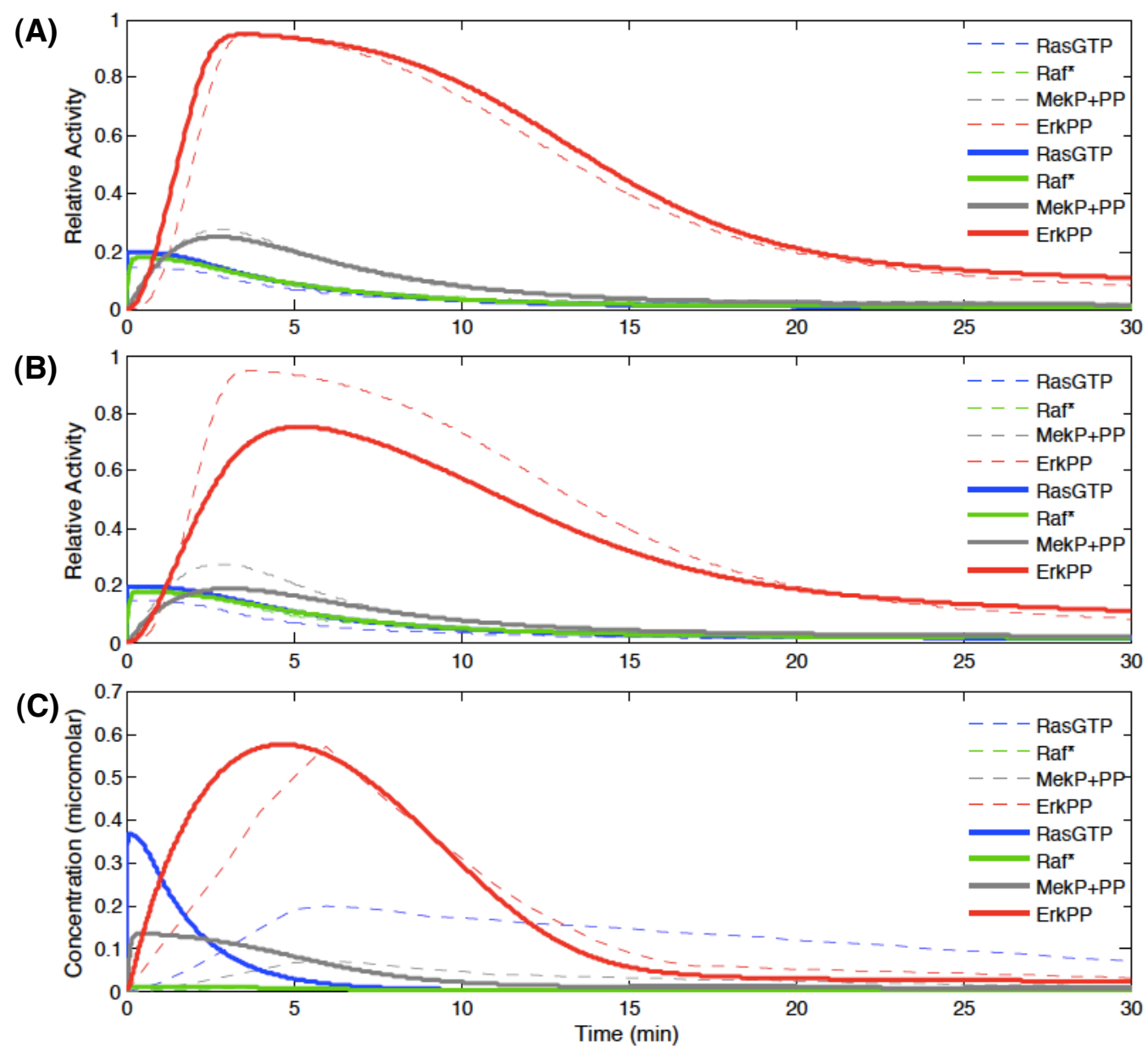

Supplement: Figure S3 — Simulated curves for the MAPK model. (A) The reduced model (solid lines) and the original Brightman and Fell model (dashed lines). (B) The reduced model with initial concentrations measured by Fujioka et al. (solid lines) and the expression data from Fujioka et al. (dashed lines). (C) The reduced model with initial concentrations measured by Fujioka et al. plus parameter sets optimized according to the FRET data measured by Fujioka et al. (solid lines) and the expression data from Fujioka et al [26]. (PDF) [file pcbi.1002738.s003.pdf]

Figure S4

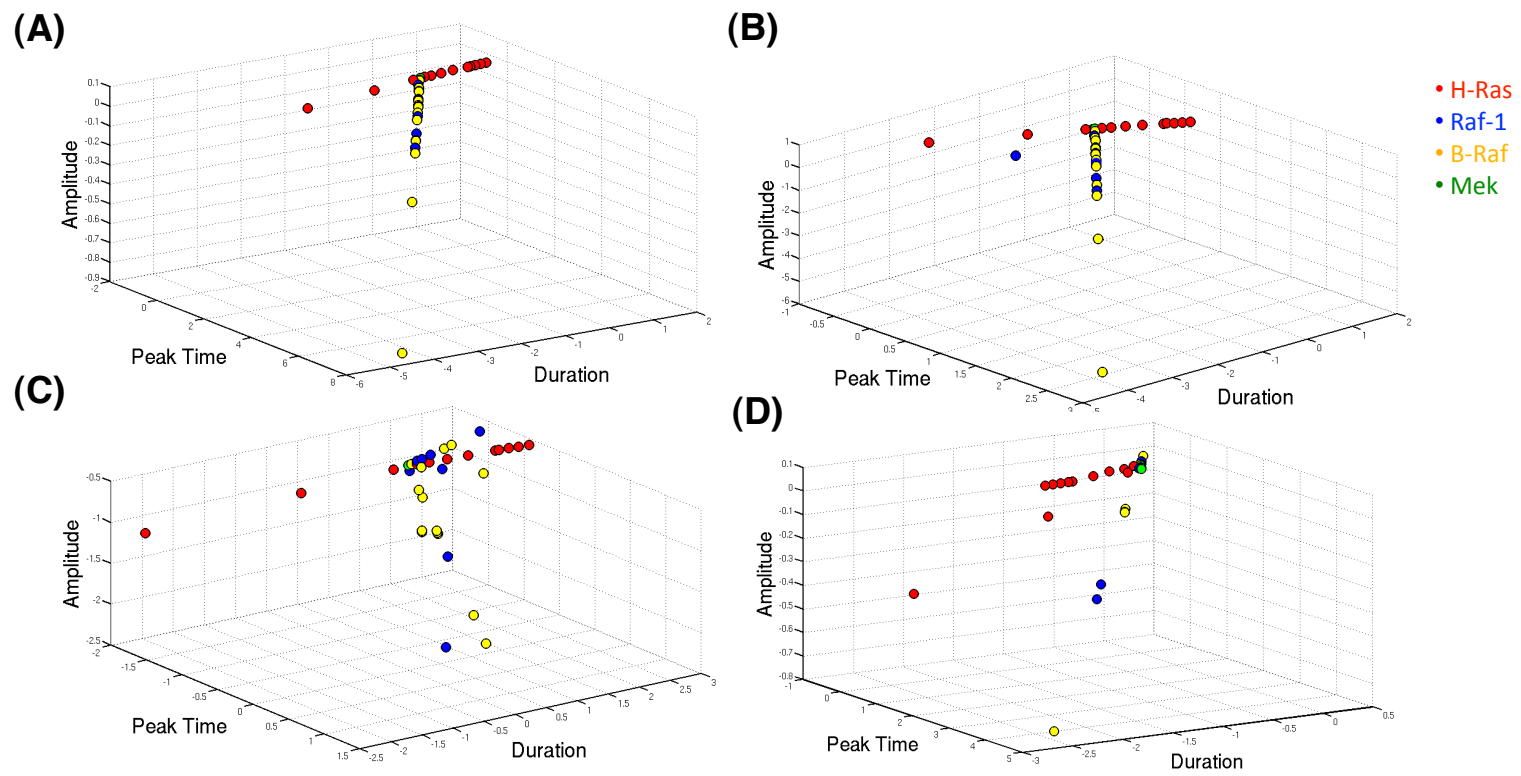

Supplement: Figure S4 — The SIF scores of the mutations in the MAPK model considering conformational ensembles. (A) The reduced model; (B) the reduced model with initial conditions from Fujioka et al.; (C) the reduced model with initial conditions from Fujioka et al. and parameters optimized by fitting to the time course data in Fujioka et al.; (D) the original non-reduced model. (PDF) [file pcbi.1002738.s004.pdf]

Figure S5

(A)

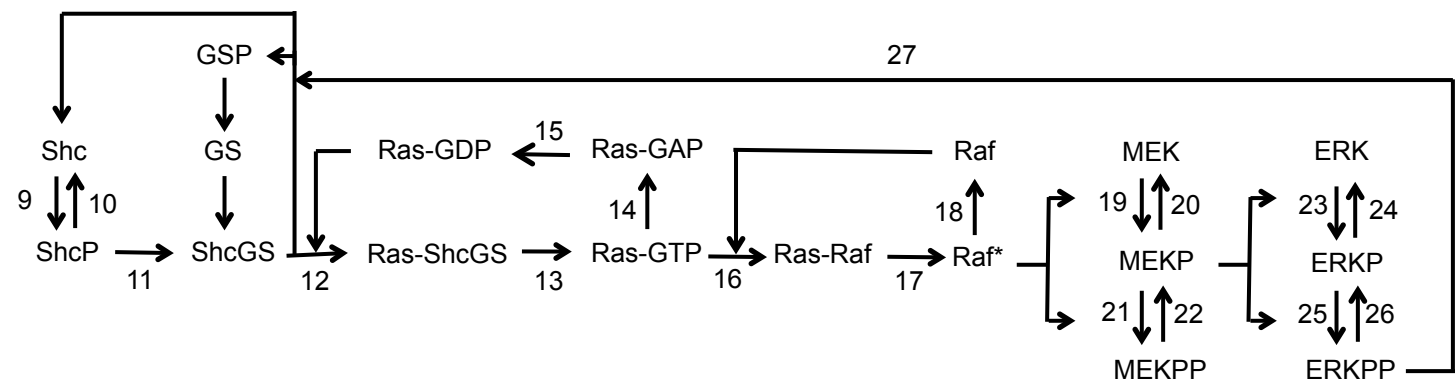

(B)

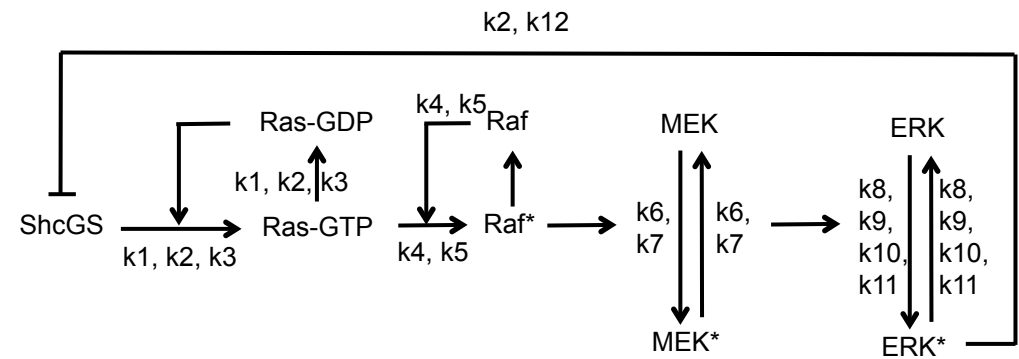

Supplement: Figure S5 — An overall structure of the original and reduced model. (A) The original non-reduced model and (B) the reduced model. (PDF) [file pcbi.1002738.s005.pdf]

Figure S6

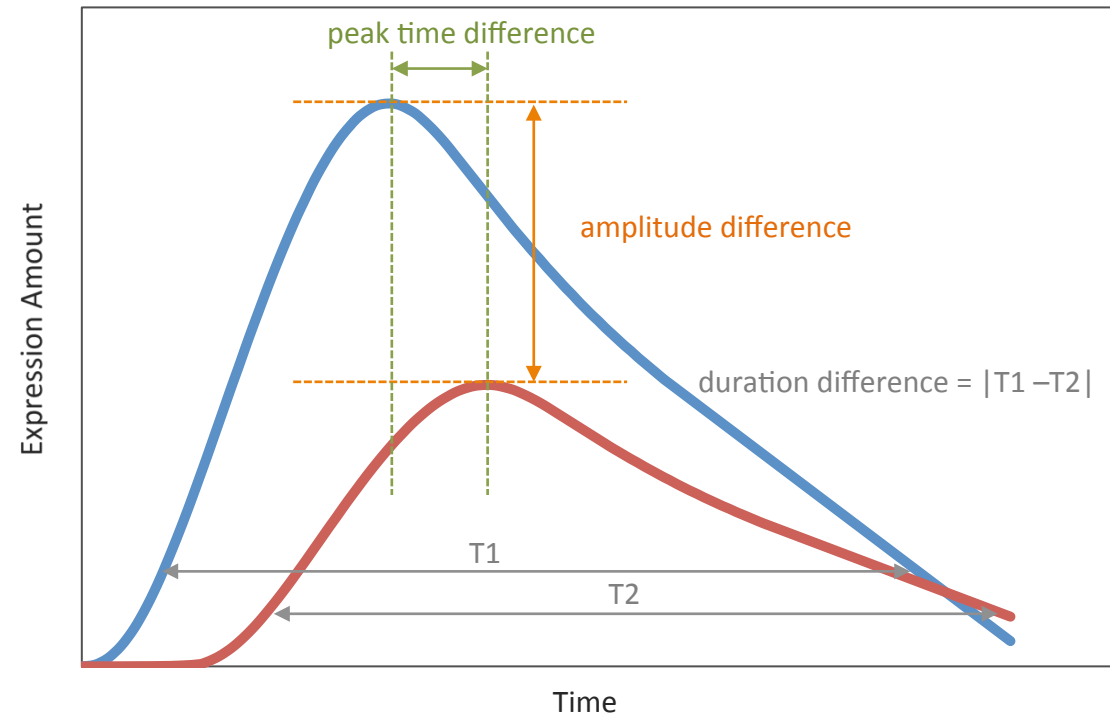

Supplement: Figure S6 — The three measurements used to quantify the difference between two proteins expression curves. (PDF) [file pcbi.1002738.s006.pdf]

Figure S7

(A)

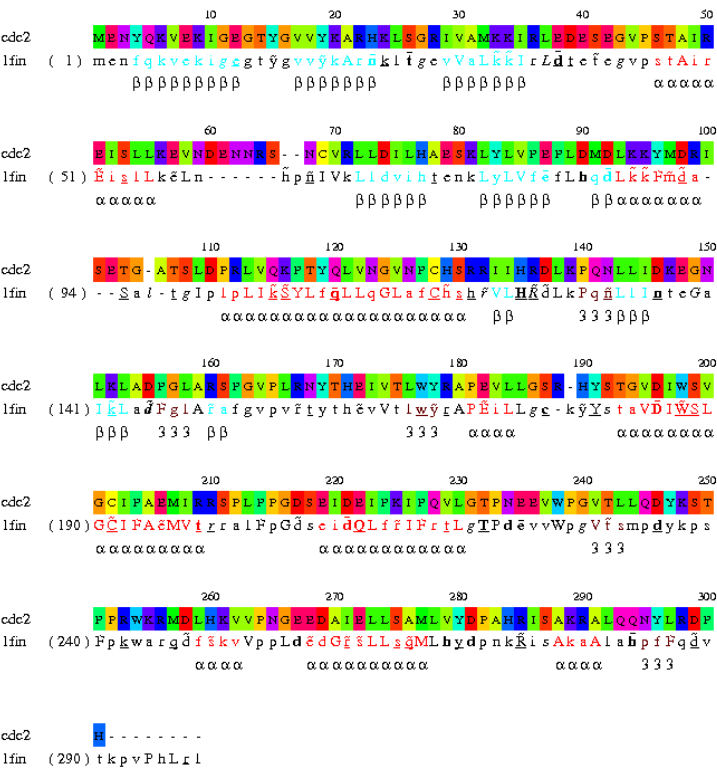

(B)

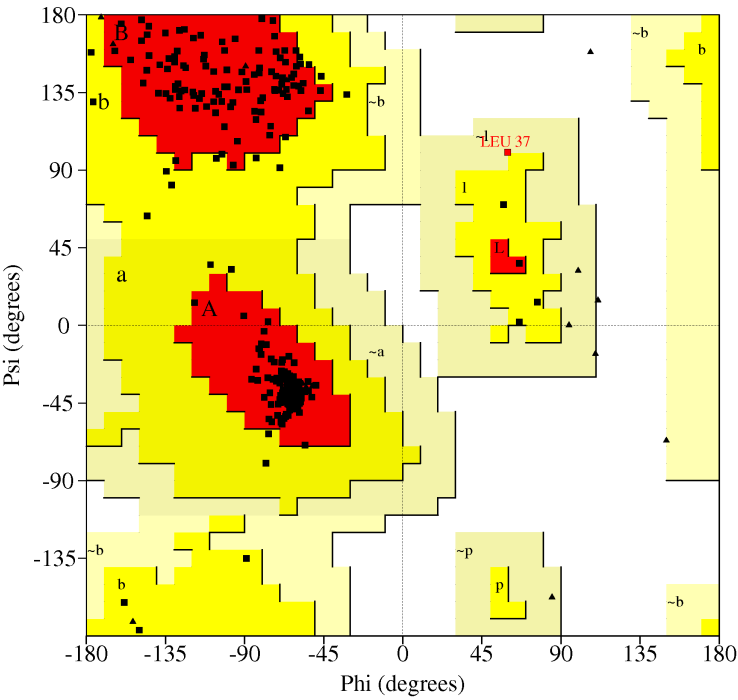

Supplement: Figure S7 — Structural analysis of Cdk1 model. (A) The alignment of Cdk1 sequence and the template structure PDB: 1FIN. The structure features of the template are shown in the JOY [52] format: each alpha helix is indicated in red, beta strand in blue and 310 helix in maroon. Solvent accessible residues are shown in lower case, solvent inaccessible in upper cases. Residues hydrogen bonded to main-chain amide groups are shown in bold style; those hydrogen bonded to main-chain carbonyl groups are underlined. Positive phi torsion angle in italic style; disulfide bonds are indicated with cedilla (B) Ramachandran plot of the Cdk1 modeled structure. Residues that have a less favorable but generally acceptable backbone conformation are highlighted in red. (PDF) [file pcbi.1002738.s007.pdf]

Figure S8

(A)

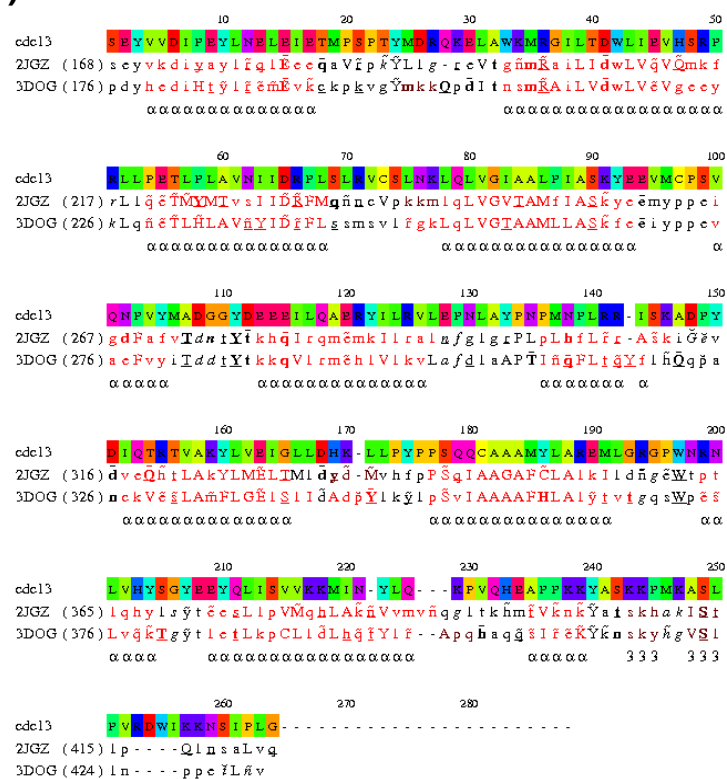

(B)

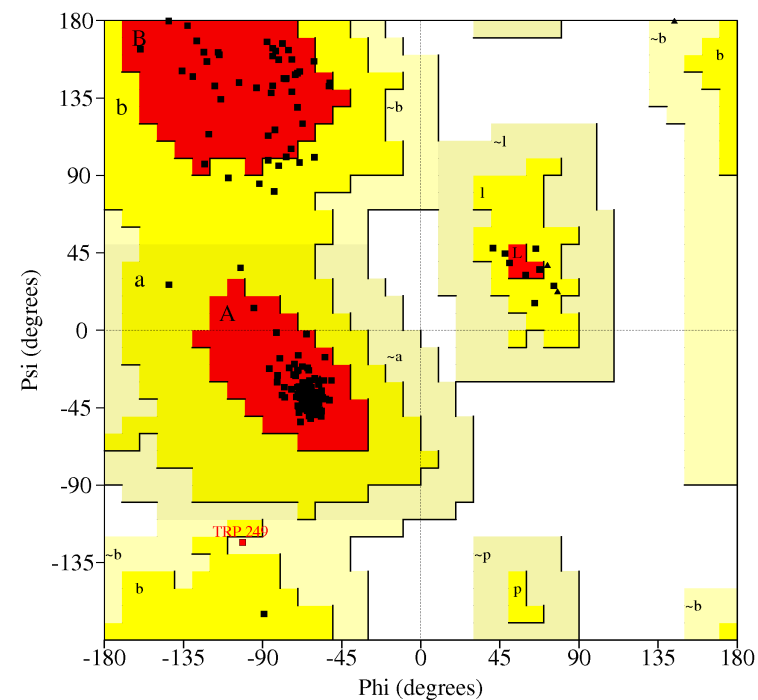

Supplement: Figure S8 — Structural analysis of CycB model. (A) The alignment of CycB sequence and the template structures PDB: 2JGZ and 3DOG. The structural features of the template are shown in the JOY [52] format, as explained in the legend of Figure S7. (B) Ramachandran plot of the CycB modeled structure. Residues that have less favorable but generally acceptable backbone conformation are highlighted in red. (PDF) [file pcbi.1002738.s008.pdf]

Figure S9

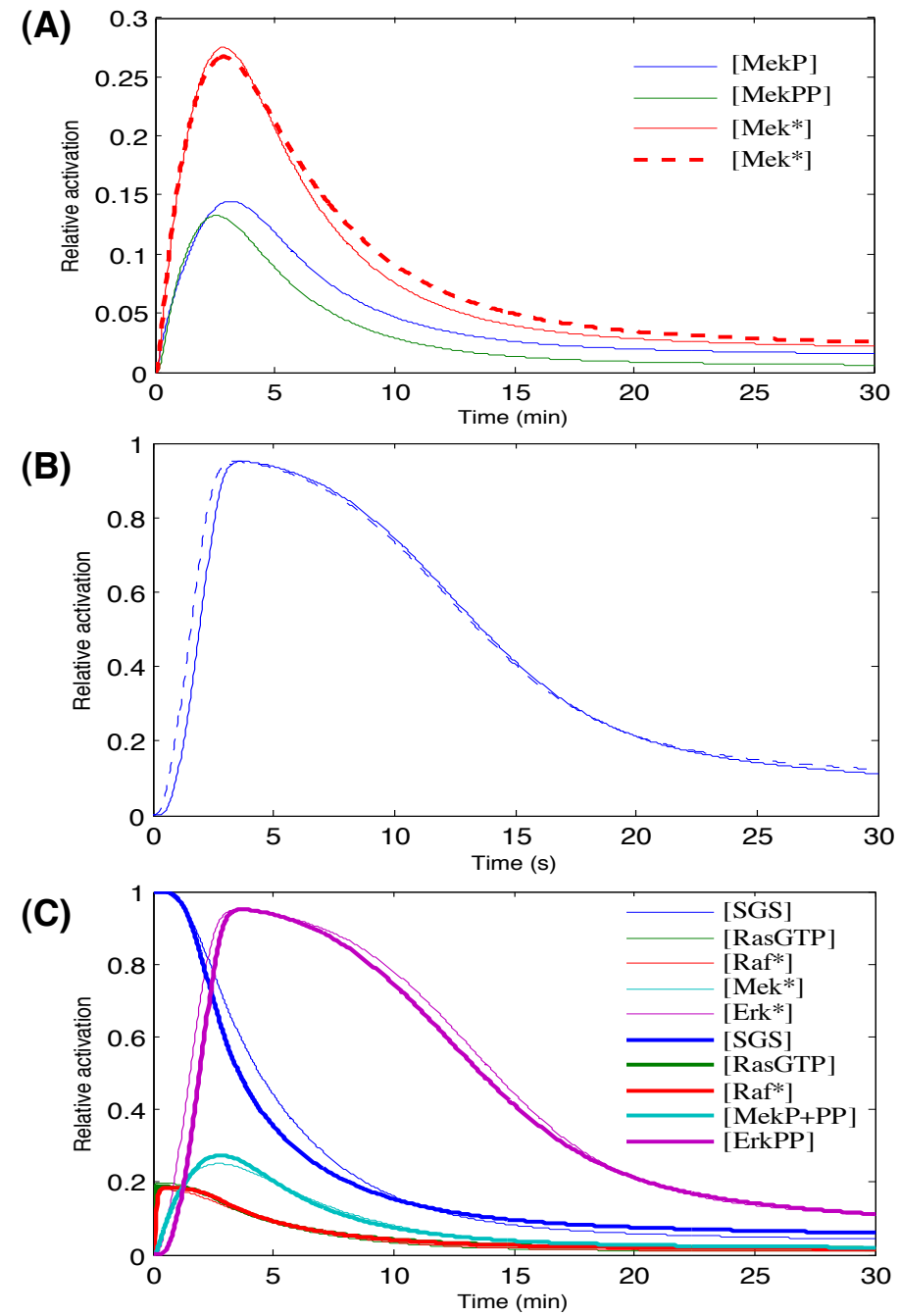

Supplement: Figure S9 — Simulated curves of the reduced and original models. (A) Comparison of the relative activation of Mek, for the Brightman and Fell model (solid lines), and the situation where the Mek activation is replaced by Eqn. 21 (dashed line). Here note [Mek*] = [MekP]+[MekPP]. (B) Comparison of the relative activation (concentration of active form, divided by initial concentration of protein) of ErkPP between the original Brightman and Fell model (solid line) and the simplified version in which the Erk activation is replaced by Eqn. 24 (dashed line). (C) Comparison of the relative activation between the Brightman and Fell (2000) model (heavy lines) and the equivalent simplified model (light lines). (PDF) [file pcbi.1002738.s009.pdf]

Figure S10

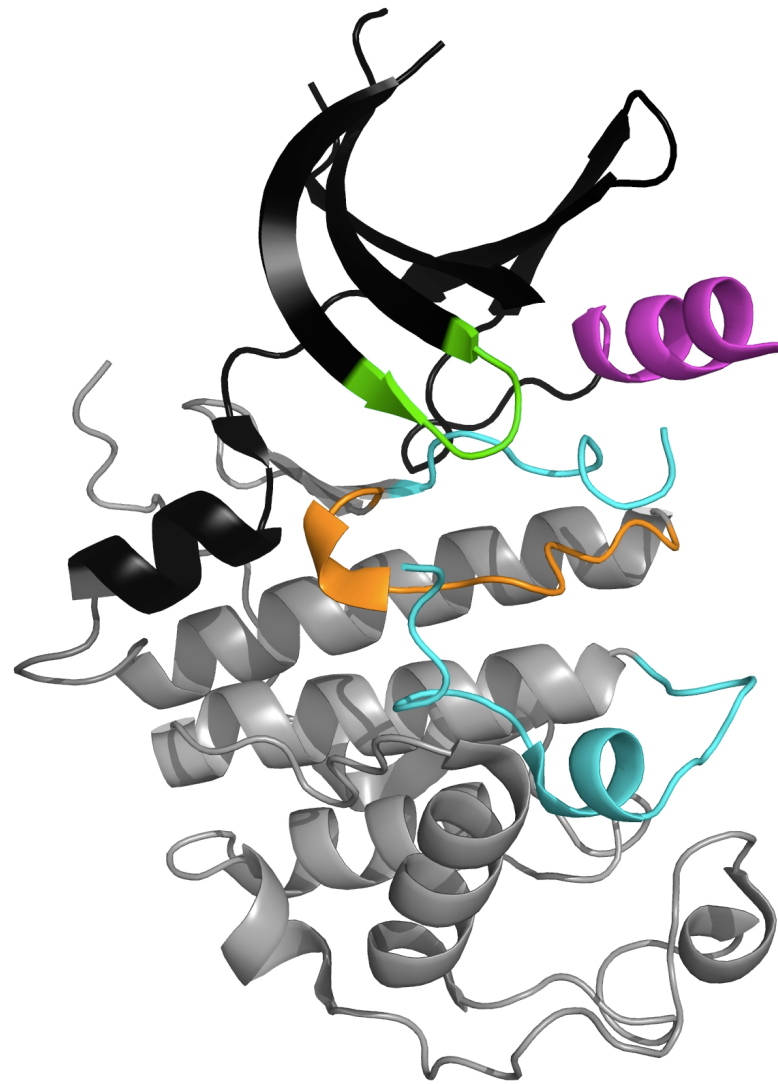

Supplement: Figure S10 — Three-dimensional structure of a kinase. The G-rich loop is colored in green; the C-alpha helix is colored in magenta; the catalytic loop is colored in orange; the activation loop is colored in cyan. The N-lobe region is colored in black while the C-lobe is colored in grey. (PDF) [file pcbi.1002738.s010.pdf]
