# Supplementary material for: A Structural Systems Biology Approach for Quantifying the Systemic Consequences of Missense Mutations in Proteins
Source: PLoS Comput Biol. 2012 Oct 18;8(10):e1002738. doi: 10.1371/journal.pcbi.1002738 (PMC3475653; doi:10.1371/journal.pcbi.1002738)
Supplement: Table S1 — Mutations and model parameters associated with neuro-cardio-facial-cutaneous syndrome. (DOC) [file pcbi.1002738.s011.doc]

**Table S1**. Mutations and model parameters associated with neuro-cardio-facial-cutaneous syndrome

| Protein | Mutationa | Phenotype | References | Impact Type b | Corresponding parameter | ∆∆G (kcal/mol) c | CSpi d | | | SIF | | |
| --- | --- | --- | --- | --- | --- | --- | --- | --- | --- | --- | --- | --- |
|  |  |  |  |  |  |  | Peak difference | Duration change | Peak time change | Peak difference | Duration change | Peak time change |
| H-Ras | G12A | CS |  | F | k2 | 2.28 | 0.013 | 0.85 | 0.04 | 0.030 | 1.938 | 0.091 |
|  | G12E | CS |  | F | k2 | 2.52 | 0.013 | 0.85 | 0.04 | 0.033 | 2.142 | 0.101 |
|  | G12C | CS |  | F | k2 | 1.89 | 0.013 | 0.85 | 0.04 | 0.025 | 1.607 | 0.076 |
|  | G12S | CS |  | F | k2 | 3.11 | 0.013 | 0.85 | 0.04 | 0.040 | 2.644 | 0.124 |
|  | G12V | CS |  | F | k2 | 2.78 | 0.013 | 0.85 | 0.04 | 0.036 | 2.363 | 0.111 |
|  | G12D | CS |  | F | k2 | 4.69 | 0.013 | 0.85 | 0.04 | 0.061 | 3.987 | 0.188 |
|  | G13C | CS |  | F | k2 | 3.51 | 0.013 | 0.85 | 0.04 | 0.046 | 2.984 | 0.140 |
|  | G13D | CS |  | F | k2 | 4.93 | 0.013 | 0.85 | 0.04 | 0.064 | 4.191 | 0.197 |
|  | T58I | CS | [6] | F | k2 | -2.36 | 0.013 | 0.85 | 0.04 | -0.031 | -2.006 | -0.094 |
|  | K117R | CS |  | S | [RasGDP]0 e | 0.76 | -0.02 | -0.83 | -0.03 | -0.015 | -0.631 | -0.023 |
|  | A146T | CS | [5] | S | [RasGDP]0 | 4.16 | -0.02 | -0.83 | -0.03 | -0.083 | -3.453 | -0.125 |
|  | A146V | CS | [6] | S | [RasGDP]0 | 1.58 | -0.02 | -0.83 | -0.03 | -0.032 | -1.311 | -0.047 |
| Raf-1 | D486N | NS | [7] | F | k4 | -1.43 | 0.06 | 0.38 | -0.55 | -0.086 | -0.543 | 0.787 |
|  | D486G | NS | [7] | F | k4 | 0.02 | -0.08 | -0.42 | 0.71 | -0.002 | -0.008 | 0.014 |
|  | T491I | NS | [7] | F | k4 | -0.23 | -0.08 | -0.42 | 0.71 | 0.018 | 0.097 | -0.163 |
|  | T491R | NS | [7] | F | k4 | 1.41 | -0.08 | -0.42 | 0.71 | -0.113 | -0.592 | 1.001 |
|  | S612T | NS | [7] | S | [Raf-1]0 | 0.12 | -0.08 | -0.42 | 0.71 | -0.010 | -0.050 | 0.085 |
|  | L613V | NS | [7] | S | [Raf-1]0 | 0.68 | -0.08 | -0.42 | 0.71 | -0.054 | -0.286 | 0.483 |
| B-Raf | S466A | CFCS | [8] | F | k4 | 0.43 | -0.08 | -0.42 | 0.71 | -0.034 | -0.181 | 0.305 |
|  | F467S | CFCS | [8] | F | k4 | 2.04 | -0.08 | -0.42 | 0.71 | -0.163 | -0.857 | 1.448 |
|  | G468E | CFCS |  | F | k4 | 13.99 | -0.08 | -0.42 | 0.71 | -1.119 | -5.876 | 9.933 |
|  | L484F | CFCS |  | F | k4 | 0.79 | -0.08 | -0.42 | 0.71 | -0.063 | -0.332 | 0.561 |
|  | K498E | CFCS | [8,9] | S | [B-Raf]0 | 0.28 | -0.08 | -0.42 | 0.71 | -0.022 | -0.118 | 0.199 |
|  | K498N | CFCS |  | S | [B-Raf]0 | 0.52 | -0.08 | -0.42 | 0.71 | -0.042 | -0.218 | 0.369 |
|  | E500G | CFCS | [8,9] | F | k4 | 2.11 | -0.08 | -0.42 | 0.71 | -0.169 | -0.886 | 1.498 |
|  | E500K | CFCS | [8,9,10] | F | k4 | 2.53 | -0.08 | -0.42 | 0.71 | -0.202 | -1.063 | 1.796 |
|  | L524P | CFCS |  | S | [B-Raf]0 | 1.05 | -0.08 | -0.42 | 0.71 | -0.084 | -0.441 | 0.746 |
|  | W530C | NS | [10] | S | [B-Raf]0 | 2.98 | -0.08 | -0.42 | 0.71 | -0.238 | -1.252 | 2.116 |
|  | N580D | CFCS | [8,9] | F | k4 | 3.59 | -0.08 | -0.42 | 0.71 | -0.287 | -1.508 | 2.549 |
|  | F594L | CFCS |  | F | k4 | 1.58 | -0.08 | -0.42 | 0.71 | -0.126 | -0.664 | 1.122 |
|  | G595V | CFCS | [8] | F | k4 | 3.55 | -0.08 | -0.42 | 0.71 | -0.284 | -1.491 | 2.521 |
|  | L596V | NS |  | F | k4 | 1.48 | -0.08 | -0.42 | 0.71 | -0.118 | -0.622 | 1.051 |
|  | T598R | CFCS |  | F | k4 | 0.07 | -0.08 | -0.42 | 0.71 | -0.006 | -0.029 | 0.050 |
|  | D637E | CFCS |  | F | k4 | 4.25 | -0.08 | -0.42 | 0.71 | -0.340 | -1.785 | 3.018 |
|  | Q708R | CFCS |  | S | [B-Raf]0 | 0.95 | -0.08 | -0.42 | 0.71 | -0.076 | -0.399 | 0.675 |
| Mek-1 | F53S | CFCS | [8] | S | [Mek]0 | 2.79 | 0.0001 | 0.0001 | 0.0001 | 0.0003 | 0.0003 | 0.0003 |
|  | P124Q | CFCS |  | S | [Mek]0 | 3.13 | 0.0001 | 0.0001 | 0.0001 | 0.0003 | 0.0003 | 0.0003 |
|  | Y130C | CFCS | [8] | S | [Mek]0 | 3.84 | 0.0001 | 0.0001 | 0.0001 | 0.0004 | 0.0004 | 0.0004 |
|  | Y130H | CFCS |  | S | [Mek]0 | 2.23 | 0.0001 | 0.0001 | 0.0001 | 0.0002 | 0.0002 | 0.0002 |
|  | E203Q | CFCS |  | S | [Mek]0 | 0.92 | 0.0001 | 0.0001 | 0.0001 | 0.0001 | 0.0001 | 0.0001 |

a The residue number is shown as it is in the crystal structures – H-Ras: 121P (PDB code); Raf-1: 3LB7; B-Raf: 1UWH; Mek-1: 3EQC

b Each mutation is considered to have mainly functional (F) or structural (F) impact according to their locations in its target protein.

c For H-Ras, both the uncomplexed and complexed form (SOS-Ras) are available for evaluating the ∆∆G of missense mutations. For the two ∆∆G values calculated in the complexed and uncomplexed states, the one with the maximum absolute value is used for calculating the SIF score. For Raf-1, B-Raf and Mek-1, only their monomeric structures are used to calculate ∆∆G (the interaction between HRas-Raf1, Raf1-Mek1 and BRaf-Mek1 kinase domains are so transient and it is difficult to solve or model the complexed form). All the uncomplexed form are monomeric structure except BRaf, which exists as a homodimer.

d For mutations that are considered to cause mainly functional impacts, their  ­was calculated as perturbation of rate constants that is associated with the activation of downstream proteins – that is, k2, k4 and k6 in the case of H-Ras, Raf-1 and Mek mutations, respectively. For mutations that are considered to cause mainly structural impacts, their  ­was calculated as perturbation of the initial conditions of the target protein. was calculated by increasing or reducing the target parameter based on the ∆∆G value by 10%. For example, if a mutation is likely to cause structural impacts and its ∆∆G suggests a reduced stability, then its  was calculated by reducing the initial condition of the target protein by 10%.

e [A]0 represents the initial concentration of protein A.

Notes:

1. This is not a comprehensive list of the NCFC mutations identified so far; mutations located in regions without structures are not discussed here.

2. ∆∆G of B-raf is estimated according to the values of Raf-1 since the endogenous concentration of B-Raf is nearly four times lower than that of Raf-1 (0.0035 and 0.013 µM, respectively ), and the expression amount of Raf-1 is already very low compared to that of Ras (0.4), Mek (1.4) and Erk (0.96) (Figure S3). A lower expression curve of B-raf is not likely to change much the parameters of the model hence we gauged the impact of B-raf by the curve and  of Raf-1.

References:

1. Aoki Y, Niihori T, Kawame H, Kurosawa K, Ohashi H, et al. (2005) Germline mutations in H-RAS proto-oncogene cause Costello syndrome. Nat Genet 37: 1038-1040.

2. Kerr B, Delrue MA, Sigaudy S, Perveen R, Marche M, et al. (2006) Genotype-phenotype correlation in Costello syndrome: HRAS mutation analysis in 43 cases. J Med Genet 43: 401-405.

3. Gripp KW, Lin AE, Stabley DL, Nicholson L, Scott CIJ, et al. (2006) H-RAS mutation analysis in Costello syndrome: genotype and phenotype correlation. Am J Med Genet A 140: 1-7.

4. Lo IF, Brewer C, Shannon N, Shorto J, Tang B, et al. (2008) Severe neonatal manifestations of Costello syndrome. J Med Genet 45: 167-171.

5. Zampino G, Pantaleoni F, Carta C, Cobellis G, Vasta I, et al. (2007) Diversity, parental germline origin, and phenotypic spectrum of de novo HRAS missense changes in Costello syndrome. Hum Mutat 28: 265-272.

6. Gripp KW, Innes AM, Axelrad ME, Gillan TL, Parboosingh JS, et al. (2008) Costello syndrome associated with novel germline HRAS mutations: an attenuated phenotype? Am J Med Genet A 146A: 683-690.

7. Pandit B, Sarkozy A, Pennacchio LA, Carta C, Oishi K, et al. (2007) Gain-of-function RAF1 mutations cause Noonan and LEOPARD syndromes with hypertrophic cardiomyopathy. Nat Genet 39: 1007-1012.

8. Rodriguez-Viciana P, Tetsu O, Tidyman WE, Estep AL, Conger BA, et al. (2006) Germline mutations in genes within the MAPK pathway cause cardio-facio-cutaneous syndrome. Science 311: 1287-1290.

9. Niihori T, Aoki Y, Narumi Y, Neri G, Cavé H, et al. (2006) Germline KRAS and BRAF mutations in cardio-facio-cutaneous syndrome. Nat Genet 38: 294-296.

10. Sarkozy A, Carta C, Moretti S, Zampino G, Digilio MC, et al. (2009) Germline BRAF mutations in Noonan, LEOPARD, and cardiofaciocutaneous syndromes: molecular diversity and associated phenotypic spectrum. Hum Mutat 30: 695-702.

11. Rauen KA, Tidyman WE, Estep AL, Sampath S, Peltier HM, et al. (2010) Molecular and functional analysis of a novel MEK2 mutation in cardio-facio-cutaneous syndrome: transmission through four generations. Am J Med Genet A 152A: 807-814.

12. Dentici ML, Sarkozy A, Pantaleoni F, Carta C, Lepri F, et al. (2009) Spectrum of MEK1 and MEK2 gene mutations in cardio-facio-cutaneous syndrome and genotype-phenotype correlations. Eur J Hum Genet 17: 733-740.

13. Nyström AM, Ekvall S, Berglund E, Björkqvist M, Braathen G, et al. (2008) Noonan and cardio-facio-cutaneous syndromes: two clinically and genetically overlapping disorders. J Med Genet 45: 500-506.
