# Supplementary material for: A Structural Systems Biology Approach for Quantifying the Systemic Consequences of Missense Mutations in Proteins
Source: PLoS Comput Biol. 2012 Oct 18;8(10):e1002738. doi: 10.1371/journal.pcbi.1002738 (PMC3475653; doi:10.1371/journal.pcbi.1002738)
Supplement: Text S1 — Additional information of the in silico MAPK model. This file describes the parameter values optimized based on the FRET data measured by Fujioka et al. (DOC) [file pcbi.1002738.s012.doc]

**Text S1. Additional information of the *in silico* MAPK model**

To check the robustness of inferring systemic impact, we derived alternative parameter sets based on the FRET data measured by Fujioka *et al.* :

Parameter set 1 (derived from initial concentrations of ShcGS, Ras, Raf, Mek and Erk measured by Fujioka *et al*.):

*Initial concentrations (μM):*

*ShcGS = 0.4; RasGDP = 0.4; RasGTP = 0; Raf = 0.013, Raf* = 0; Mek = 1.4; Mek* = 0; Erk = 0.96; Erk* = 0*

*Rate constants (μM min^-1^):*

*c_2_ = 38.5; c_6_ = 2.73; c_8_ = 0.51; c_10_ = 15; c_12_ = 3.15*

*Rate constants (μM):*

*c_7_ = 0.35; c_9_ = 0.77; c_11_ = 0.03*

*Rate constants (min^-1^):*

*c_1_ = 69; c_3_ = 14; c_4_ = 107.7; c_5_ = 0.78*

Parameter set 2 (optimized based on the time course data from Fujioka *et al.*, with initial concentrations of ShcGS, Ras, Raf, Mek and Erk measured by Fujioka *et al*.):

*Initial concentrations (μM):*

*ShcGS = 0.4; RasGDP = 0.4; RasGTP = 0; Raf = 0.013, Raf* = 0; Mek = 1.4; Mek* = 0; Erk = 0.96; Erk* = 0*

*Rate constants (μM min^-1^):*

*c_2_ = 0.82; c_6_ = 5.02; c_8_ = 20.73; c_10_ = 33.9; c_12_ = 0.54*

*Rate constants (μM):*

*c_7_ = 70.3; c_9_ = 20.27; c_11_ = 0.96*

*Rate constants (min^-1^):*

*c_1_ = 27.32; c_3_ = 2.14; c_4_ = 187.15; c_5_ = 16.08*

References:

1. Fujioka A, Terai K, Itoh RE, Aoki K, Nakamura T, et al. (2006) Dynamics of the Ras/ERK MAPK cascade as monitored by fluorescent probes. J Biol Chem 281: 8917-8926.
