# Supplementary material for: A Structural Systems Biology Approach for Quantifying the Systemic Consequences of Missense Mutations in Proteins
Source: PLoS Comput Biol. 2012 Oct 18;8(10):e1002738. doi: 10.1371/journal.pcbi.1002738 (PMC3475653; doi:10.1371/journal.pcbi.1002738)
Supplement: Text S2 — Characterizing structural and functional mutations. This file discusses the separation of structural and functional mutations studied in this work. (DOC) [file pcbi.1002738.s013.doc]

**Text S2. Characterizing structural and functional mutations**

One of the objectives of this study is to determine how the kinetic behavior of a sequence of enzymes composing a pathway can be explained in terms of the properties of individual isolated enzymes. Since the only way to perturb the activity of an enzyme in a system without perturbing anything else, as discussed in , is through perturbing protein concentrations, the systemic impact of a missense mutation that affects mainly structure but not function of a protein can be estimated directly by varying concentration-related parameters. In the yeast G2-M model, this is related to perturbation on rate constants k_s_ and k_d_, which regulates the concentration of Cdk1, CycB and MPF, respectively. These parameters are used to describe the temporal synthesis or degradation of proteins during the cell cycle. In the human ERK model, this is related to perturbation of the initial concentration of Ras, Raf and Mek, assuming that these kinases are always available in the cell and missense mutations are likely to affect their existing concentrations before the signaling transduction mechanism is triggered. Perturbations of the above parameters are estimated through protein stability change (∆∆G), which affect the lifetime of a protein and thus are likely to change its concentration in the cell .

For mutations that are located within or on the peripheral of a protein active site, we estimate their impact on the related Michaelis constants but not the rate-limiting constant (which describes the rate of bound substrate conversion to product). For example, in the yeast G2-M model, we perturbed J_iwee_ and J_25_ that regulate MPF's ability to inactivate wee1 and to activate cdc25, respectively. In the human ERK model, we perturbed c_7_ and c_9_ that regulate the activation and inactivation of Erk, respectively. The degree of perturbation on the Michaelis constants (K_M_) can be estimated from the mutated protein's free energy change ∆∆G, via its relationship with the disassociation constant K_d_. For example, considering the following catalytic reaction:

where E stands for enzyme, S for substrate and P for product.

The Michaelis constant K_M_ can be approximated as K_d_ when k_2_ << k_-1_

Since , the perturbation on K_d_ can be calculated as:

where MT and WT refer to mutant type and wild type protein, respectively. R is the gas constant (kcal K^-1^ mol^-1^) and T is the absolute temperature (K).

In reality, many protein-protein interactions do not suffice the condition k_2_ << k_-1_, and therefore the impact on K_d_ is not a precise measurement of the potential impact on Michaelis constants. However, it can still be useful for approximating the rank or classification of mutations, and is suitable for our case.

Here we consider mutations in the glycine-rich loop, alpha-C helix, catalytic loop and activation loop of a host kinase as likely to cause functional impacts, and consider mutations in the remaining regions as likely to cause structural impacts (see Figure S10 for the functional sites in a kinase). Also, none of the structural mutations are located in the hinge connecting the N-lobe and C-lobe, and thus are not likely to affect the structure of the functional sites through impeding the relative movements between the two lobes. Finally, all the functional mutations are located at protein surfaces and have few interactions, if any, with their neighbouring residues. Therefore the likelihood for them to cause structural effects is low. As for the only non-kinase protein CycB, the definition of the structural mutations is clear since they are located in the hydrophobic core of the protein.

References:

1. Cornish-Bowden A (2004) Fundamentals of Enzyme Kinetics: Portland Press Ltd.

2. Stack JH, Whitney M, Rodems SM, Pollok BA (2000) A ubiquitin-based tagging system for controlled modulation of protein stability. Nature Biotechnology 18: 1298-1302.
