# Supplementary material for: A Structural Systems Biology Approach for Quantifying the Systemic Consequences of Missense Mutations in Proteins
Source: PLoS Comput Biol. 2012 Oct 18;8(10):e1002738. doi: 10.1371/journal.pcbi.1002738 (PMC3475653; doi:10.1371/journal.pcbi.1002738)
Supplement: Text S3 — Formulating the reduced ODEs based on the Brightman and Fell model. This file describes the steps of producing the reduced model. (DOC) [file pcbi.1002738.s014.doc]

**Text S3. Formulating the reduced ODEs based on the Brightman and Fell model**

The following sections describe the process of simplifying the original Brightman and Fell model (2000). Rate constants are numbered as shown in Figure 1 of the original paper (also shown in Figure S5A).

**3.1 Simplifying RasGTP decay associated with GAP**

In Brightman and Fell (2000), the reaction that describes the decay of the active form RasGTP by GAP, through an intermediate complex, is

(1)

The reaction was represented by the differential equations

(2)

However, if the concentration of the intermediate complex is in a quasi-steady-state (as in the classic analysis of Briggs and Haldande (1925)), such that, for example,

(3)

then

(4)

(5)

Conservation of the GAP protein

$[GAP]+[RasGAP]=[GAP]_{0}$ (6)

where [GAP]_0_ is the initial concentration of GAP, then allows us to rearrange Eqns. 4 and 5 to yield

(7)

where is the Michaelis constant of the reaction.

We can further simplify the reaction, here, by noting that the concentration of RasGTP in the original model does not exceed 4,000 (where all concentrations are defined throughout this section as molecules per cell), whereas the Michaelis constant for this reaction is 156,000. In the limit

(8)

Eqn. 7 reduces to

(9)

representing the decay reaction

(10)

with a rate constant

(11)

Using the parameters min^-1^, min ^-1^, min ^-1^, and $[\text{GAP}]_{0}=15,000$allows us to derive the decay rate $c_{1}=69$ min^-1^.

**3.2 Simplifying Raf activation**

As with the RasGTP decay in the original model, the activation of Raf is thought to proceed through the intermediate complex

(12)

which was represented by a set of four differential equations, analogous to Eqn. 2 above. Assuming the equilibrium of the intermediate complex allows us to proceed immediately to a single reaction equation

(13)

Here, the magnitude of the Michaelis constant, 25,000, is comparable to the initial concentration of Raf, [Raf]_0_ = 10,000. The simplifying assumption that was made for the decay of RasGTP is not appropriate here. However, [Raf] varies by less than 30% of its initial magnitude, during the entire reaction. Thus, the denominator of Eqn. 16 does not change significantly during the course of the reaction. More formally, if [Raf]_0_ is the initial concentration of Raf, then Eqn. 13 is equal to

$\frac{d\text{[Raf]}}{dt}=\frac{-c_{2}\text{[Raf][RasGTP]}}{1+\delta_{R}}$ (14)

with activation rate constant

(15)

and a small correction term

(16)

which never exceeds 0.1 in magnitude during the entire reaction. We thus take the limit $\left|\delta_{R}\right|\ll1$, and obtain the simplified activation reaction

(17)

represented by the second order rate equation

$\frac{d\text{[Raf]}}{dt}=-c_{2}\text{[Raf][RasGTP]}$ (18)

Using the parameters min^-1^, min^-1^, min^-1^, and $[Raf]_{0}=10,000$ allows us to derive the appropriate activation rate constant min^-1^.

**3.3 Simplifying Raf de-activation**

In the original model Raf* deactivation occurs through the Michaelis-Menton decay reaction

(19)

where 97,000 min^-1^ and 6,000. The peak value of [Raf*] is 1,800 (initial concentration of Raf is 10,000). Proceeding similarly to the Raf activation case, the error in a simplification to a first-order rate equation is minimized by replacing the variable [Raf*] in the denominator of Eqn. 19 by a representative average value <Raf*>, here taken to be half the peak value, *ie.* <Raf*> = 900. Thus, the decay is well approximated by the simpler form

$\frac{d\text{[Raf*]}}{dt}=-c_{3}\text{[Raf*]}$ (20)

where

(21)

Using the above values, we find 14 min^-1^.

**3.4 Simplifying the Mek sequence**

The Mek protein can be activated by Raf* into either a phosphorylated (MekP), or bi-phosphorylated form (MekPP). In Brightman and Fell (2000), these are separately modeled, although the effects of MekP and MekPP on Erk activation are taken to be identical, as are the reaction rates for the following interconversions, $Mek\xrightleftharpoons{}MekP$ and $MekP\xrightleftharpoons{}MekPP$. The work of Schilling *et al.* on primary erythroid progenitor cells also support the separation of MekP and MekPP. On the other hand, Fujioka *et al*. (2006) effectively modeled the MAPK pathway with only two forms (an inactive and an active, or phosphorylated, form) of Mek, which encouraged us to explore the possibility of reducing the mono- and bi-phosphorylated states of Mek in Brightman and Fell (2000) into one active form. As shown in the following, the reduction has little effect on simulating the overall amount of the two active forms of Mek as simulated by the Brightman and Fell model. Also, the sensitivity result of perturbing the initial concentration of Raf, Mek and Erk is in line with Brightman and Fell (Figure 4) and Schilling (2009). Hence a very simple model is feasible in this case.

The original Mek reactions are activated by Raf*

(22)

where

The reactions are then represented by the differential equations

(23)

This can be immediately simplified by noting that 9,000, while the initial concentration of Mek is 360,000. At their peaks, each of MekP and MekPP has a concentration of approximately 50,000, while 600,000. Thus, assuming [MekP] and [MekPP] , and [Mek] gives

(24)

To simplify further, we introduce the concentration of active proteins, [Mek*] = [MekP] + [MekPP], and ratio $a=$ [MekPP]/[MekP]. Then,

$\frac{d\text{[Mek]}}{dt}=-\frac{d\text{[Mek*]}}{dt}=-c_{4}\text{[Raf*]}+c_{5}\text{[Mek*]}$ (25)

where 50 min^-1^, and

(26)

Superficially, we note that $a$ is approximately 1 (*ie.* there are equal concentrations of MekP and MekPP) throughout the reaction. More formally, if the concentrations of MekP and MekPP are in equilibrium with each other, then, from Eqn. 24, one can show

. (27)

At their peak values of [Raf*] = 1800, [MekP] = 52,000, and using 50, 9000, *V*_20_ = 920,000, and 600,000, one then predicts $a=$ 0.96, and 0.78 min^-1^. For long times, this linearization will tend to slightly under-predict the decay rate. However, as shown in Figure S9A, using this value gives good agreement between the simplified and full models, especially during peak activation.

**3.5 Simplifying Erk activation**

The Erk activation is similar to that of Mek in a way that MekP or MekPP can activate it into a mono- or bi-phosphorylated form, respectively. Again, various authors differ on the importance of these forms, and we have tried to explore the possibility of reducing ErkP and ErkPP into one single active form based on Brightman and Fell (2000).

In the original model, the Erk reactions are activated by [Mek*] = [MekP] + [MekPP]:

(28)

where

These reaction are represented by the differential equations

(29)

Here, briefly, we find a separation of time scales, between activation and decay, and exploit this to simplify the model.

We first note that during activation ErkP acts as an intermediate reaction step. During the activation phase, it maintains low concentrations, relative to both the initial concentration of Erk, and the peak activation concentration of ErkPP. If a molecule of ErkP is created, it rapidly either decomposes, or is bi-phosphorylated. The relative probability of a successful to an aborted activation is

(30)

As long as there is sufficient Mek* to drive the reaction, this probability is high. For example, at the peak of activation, if a molecule of Erk is phosphorylated, then it has a 95% chance of rapidly bi-phosphorylating. By making the approximation that , and treating ErkP as an intermediate reaction product, we can approximate the full reaction by

$\frac{d\text{[Erk]}}{dt}=-\frac{d\text{[Erk*]}}{dt}=-\frac{c_{6}\text{[Erk][Mek*]}}{c_{7}+\text{[Erk]}}+\frac{c_{8}\text{[Erk*]}}{c_{9}+\text{[Erk*]}}$ (31)

where we have left the slower decay of the active form (by unspecified dephosphorylation enzymes ) unchanged. Here, 8.3 min^-1^, 90,000, 400,000 min^-1^, and 600,000. Finally, we note that when [Mek*] is small, the relative error in this approximate activation rate can become large - however, as the activation rate is itself small under such conditions, the absolute error remains small. Functionally, there appear to be few differences between modeling three types of Erk, and only considering an active, and inactive form. As shown in Figure 9B, the only practical change in simplifying the ErkP equations is to advance the expression curve of ErkPP slightly, without changing its shape.

**3.6 Simplifying the feedback, and Ras activation.**

It remains to consider the feedback of Erk* on Shc-Grb-Sos (ShcGS), and the activation of Ras by ShcGS. Erk* acts on ShcGS to produce a long-lived inert form of phosphorylated GS. These reactions are

(32)

which are represented in differential equations by

(33)

As was done with the decay of Ras, the activation of Ras can be well approximated by the Michaelis-Menton rate equation

$\frac{d\text{[RasGTP]}}{dt}=\frac{c_{10}\text{[SGS]}_{a}\text{[RasGDP]}}{c_{11}+\text{[RasGDP]}}$ (34)

where 15 min^-1^, and is the remaining active concentration of ShcGS (*ie.* in the above terms, ). This is the case when the concentration of the reaction complex is taken to be in a quasi-steady-state. Using 0.0163 min^-1^, 10, and 15 allows evaluation of 1,530.

We turn finally to the negative feedback cycle, which deactivates ShcGS. Here, we can first immediately note that as 600,000, whereas the initial concentration of ShcGS is only 20,000,

(35)

We now simplify further by noting that for these reaction kinetics,

(36)

for as defined above. Thus,

(37)

and

(38)

Properly, the concentration of Ras here does not include the proteins involved in the reaction complex. To account for this would require a regression of terms. However, we find that we can approximate for this by allowing for an ad-hoc correction to the decay term, taking . For 1.6 min^-1^ and 600,000, this predicts min^-1^.

**3.7 Summary**

We have sought to find a simple representation of the Erk pathway, which captures the essential details of the pathway dynamics, and which highlights how the pathway will change, when the efficacy, decay rates, and initial concentrations of the constituent proteins are varied. Such a model also has the benefit of attempting to minimize the effects of any uncertainty that we have in the rate constants of these dynamics; only those constants that have a significant effect on the activation have been retained. When restricted to the proteins of interest, the original model contains 15 rate equations, with 27 constants. The simplified model may be summarized by five differential equations, each modeling the activation, and decay, of one particular protein:

(39)

with the rate constants

which can be derived from the rate constants in the original model. A comparison of the simplified model, to the original Brightman and Fell model is given in Figure S9C.

References

1. Schilling M, Maiwald T, Hengl S, Winter D, Kreutz C, et al. (2009) Theoretical and experimental analysis links isoform-specific ERK signalling to cell fate decisions. Mol Syst Biol 5: 334.
2. Fujioka A, Terai K, Itoh RE, Aoki K, Nakamura T, et al. (2006) Dynamics of the Ras/ERK MAPK cascade as monitored by fluorescent probes. J Biol Chem 281: 8917-8926
